# Fully Bio-Based Thermosetting Polyurethanes from Bio-Based Polyols and Isocyanates

**DOI:** 10.3390/polym13081255

**Published:** 2021-04-13

**Authors:** Roberto Morales-Cerrada, Romain Tavernier, Sylvain Caillol

**Affiliations:** Institut Charles Gerhardt Montpellier (ICGM), University of Montpellier, CNRS, ENSCM, 34095 Montpellier, France; roberto.morales-cerrada@enscm.fr (R.M.-C.); romain.tavernier@enscm.fr (R.T.)

**Keywords:** polyurethanes, bio-based polymers, isocyanates, polyols, lysine, isocyanurate, allophanate

## Abstract

The trend towards the utilization of bioresources for the manufacturing of polymers has led industry players to bring to the market new monomers. In this work, we studied 3 polyisocyanates and 2 polyols with high renewable carbon contents, namely L-lysine ethyl ester diisocyanate (LDI), pentamethylene-diisocyanate (PDI) isocyanurate trimer, and hexamethylene-diisocyanate (HDI) allophanate as the isocyanates, as well as castor oil and polypropanediol as the polyols. These monomers are commercially available at a large scale and were used in direct formulations or used as prepolymers. Thermosetting polymers with *T_g_* values ranging from −41 to +21 °C and thermal stabilities of up to 300 °C were obtained, and the polymerization was studied using NMR, DSC, and rheology. Cured materials were also characterized using FTIR, DMA, gel content, and swelling index determinations. These high bio-based content materials can successfully be obtained and could be used as alternatives to petro-based materials.

## 1. Introduction

The polymer industry has not stopped growing since the beginning of the 20th century, however this industry still mainly relies on raw fossil materials. In fact, the production of plastic materials equaled over 360 million metric tons in 2018, whereas less than 1 wt% were manufactured from bio-based raw materials [1]. Nowadays, environmental, legislative, and economical factors are increasing the demand for more sustainable materials. Biomass feedstocks are promising resources because they significantly reduce the carbon footprint and their production process may be more energy-efficient than petroleum-based plastics [2]. Currently, a large variety of bio-based or partially bio-based plastics and resins have been reported by both academic and industry communities, such as epoxy resins [3,4,5], polyesters [6], polyamides [7], or polyurethanes.

Polyurethanes (PUs) represent an important part of the polymer market. Since they were discovered by Otto Bayer in 1937 [8], demand has continuously increased due to their excellent chemical, physical, and mechanical properties, as well as their versatility, reaching 18 Mt in 2019. In fact, they are used for many applications, such as in rigid and flexible foams, coatings, adhesives, sealants, and elastomers [9]. Although the several synthetic pathways used to obtain polyurethanes without the use of isocyanates (non-isocyanate polyurethanes—NIPUs) [10,11,12,13,14,15] are promising, regular polyurethanes are still required in most applications due to the lower reactivity of cyclocarbonates [16]. PUs are generally synthesized through the reaction of polyisocyanates with polyols. Many bio-based polyols are available in the market or are reported in the literature, however the selection of bio-based isocyanates is really limited [17]; almost all bio-based PUs are only partially bio-based, since they are synthesized from bio-based polyols and petro-based isocyanates [18]. Nevertheless, the transition toward fossil-free plastics and polymers is bringing new structures to the market for all kind of applications.

Bio-based polyols are usually synthesized from vegetables oils, carbohydrates, lignocellulose, or proteins [17,19,20,21,22]. However, only *lesquerella* and castor oils naturally contain hydroxyl moieties [23,24]. Particularly, castor oil is a non-edible vegetable oil extracted from the plant *Ricinus communis*, and it has the major advantage of containing the triglyceride of ricinoleic acid (87–90%), which possesses reactive hydroxyl groups on the fatty acid residues, with a mean value of 2.7 per unit [25,26]. Additionally, castor oil is easily available commercially and its global production reached 610,000 metric tons in 2010 [27]. Consequently, this natural oil represents an ideal alternative to chemical feedstock and is, thus, readily applicable for polyurethane synthesis [17,28,29,30,31]. Thus, we selected this vegetable oil as a reference for bio-based polyols [32,33]. We also used Velvetol^®^ H500 (aka PO3G) from WeylChem International GmbH, a polypropanediol (PPD) synthesized from renewable 1,3-propanediol, which is synthesized from bio-based glycerol. In fact, glycerol is a crucial raw material for the synthesis of several products with high added value, and its production has significantly increased during the last years, since it is a by-product of biodiesel [34]. The commercial availability of bio-based PPD was attested before 2010, and was initially produced by DuPont Corporation [35], with bio-based propanediol obtained from corn sugar fermentation. This new polyol has the great advantage of containing polyether moieties, which are greatly appreciated for the synthesis of polyurethane prepolymers. Indeed, polyether polyols accounted for 71% of polyol production in 2011 [36]. Usually, polyether-based prepolymers use either PO (propylene oxide) or EO (ethylene oxide) units, especially when the purpose is to obtain segmented PUs. Currently, these kinds of building blocks are commercially available and obtained from bioresources, which have recently attracted interest in the polyurethane community [37]. For instance, polyurethane-acrylic coatings were prepared with hexamethylenediisocyanate-based isocyanurate trimers, PPD at Mn = 650 g·mol^−1^, and different acrylates. Thermoplastic polyurethanes (TPU) were also prepared using methylenediisocyanate (MDI) prepolymer with PPD values of 1000 and 2700 g·mol^−1^ [38,39]. Several molecular weights of PPD are commercially available, for instance Mn = 500, 1000, 2000, or 2700 g·mol^−1^. We chose the Mn = 500 g·mol^−1^ grade, since it is liquid at ambient temperature and with a moderate viscosity (90–120 mPa·s at 40 °C).

Concerning polyisocyanates, only a handful of bio-based polyisocyanates have been reported in literature [18,33,40,41], with scarce commercial availability. Nevertheless, isocyanates obtained from renewable materials have also attracted the interest of manufacturers, which are developing new monomers, especially from fatty acids. In fact, General Mills Inc. [42,43], Henkel Corporation [43,44,45], and BASF Europe [24,46] have developed a series of fatty acid dimer-based isocyanates, which have been used for several applications, such as the development of ultraviolet curable optical fiber coatings. Nowadays, three of the main commercially available bio-based isocyanates are pentamethylene-diisocyanate (PDI) and its oligomers (for example the PDI isocyanurate trimer from Covestro^®^); L-lysine ethyl ester diisocyanate (LDI); and Tolonate™ X FLO 100 from Vencorex^®^ Chemicals, which is based on HDI allophanate and palm oil [47,48]. PDI isocyanurate and LDI contain maximal renewable carbon contents, whereas Tolonate™ X FLO 100 is only partially bio-based. Thus, we chose to study these monomers. LDI has mainly been reported for biomedical applications [49,50]. Hence, poly(ester-urethane)s were designed as bioabsorbable networks, based on LDI and copolyesters of ε-caprolactone and D,L-lactide, and have been studied for their biodegradation or biocompatibility [51,52]. The low toxicity of degradation products from LDI-based networks, such as L-lysine amino acid, also make this isocyanate very attractive for the production of sustainable polyurethane [53,54,55,56]. Tolonate™ X FLO 100 has been used in several research articles, mainly using vegetable-oil-derived polyols. For instance, polyurethane foams have been prepared using a poly(propyleneglycol)–Tolonate prepolymer and modified vegetable oils [57]. Castor oil and Tolonate™ X FLO 100 were used together in order to prepare waterborne polyurethane nanocomposites, using either silica or clay as the filler [58,59]. On the other hand, Desmodur^®^ eco N 7300 is synthesized by amination of glucose with ammonia, followed by reaction with phosgene to obtain PDI. Subsequently, PDI is oligomerized with a catalyst such as tri-*n*-butylphosphine to yield PDI trimers, as well as pentamers, heptamers, and higher molecular weight oligomers [60]. The formation of these oligomers decreases the NCO content and increases the viscosity of the product. Additionally, the viscosity of aliphatic isocyanurates is higher for shorter carbon chains [61], which results in a particularly high viscosity (≈9200 mPa·s at 25 °C for Desmodur eco N 7300). Nevertheless, the chemical structure of polyisocyanurates (PIR) improves the mechanical and insulation properties of the materials and makes them less flammable compared to PUs, without the use of halogens, making them attractive for many applications [62,63]. All carbons of Desmodur^®^ eco N 7300 are bio-based, except those that come from phosgene (68% bio-based carbons according to TDS results).

Fundamental knowledge, however, is still lacking in the literature about some of these building blocks, and we herein provide a study of commercially available monomers for the synthesis of bio-based polyurethanes networks. In this article, we used three bio-based or partially bio-based isocyanates and two bio-based polyols in order to obtain thermosetting polyurethanes. We performed two kind of syntheses, i.e., a one-pot synthesis, using a polyisocyanate and a polyol, as well as a two-step route, synthesizing first an isocyanate prepolymer prior to crosslinking with a chain extender. For the latter one, we used bio-based chain extenders for the curing of the isocyanate prepolymers, namely 1,3-propanediol and glycerol as difunctional and trifunctional chain extenders, respectively. Curing of the formulations was determined by DSC and monitored by rheology for gelation time determination. The new bio-based thermosetting polyurethanes were characterized using DSC, DMA, FT-IR, and TGA.

## 2. Experimental Part

### 2.1. Materials

L-Lysine ethyl ester diisocyanate (LDI, ≥97%, Alfa-Aesar, Kandel, Germany), castor oil (Sigma-Aldrich, Darmstadt, Germany), glycerol (≥99.5%, Prolabo, Paris, France), 1,3,5-trioxane (99%, Fluorochem, Hadfield, Glossop, United Kingdom), and CDCl_3_ (99.5% D, Eurisotop, Saint-Aubin, France), tetrahydrofuran (THF, HPLC grade, VWR, Radnor, PA, USA) were used as received. 1,3-Propanediol (≥98.0%, Sigma-Aldrich, Darmstadt, Germany) was distilled in the presence of K_2_CO_3_ prior to use. Desmodur^®^ eco N 7300 was kindly supplied by Covestro^®^ (Leverkusen, Germany) and used as received. Tolonate™ X FLO 100 was kindly supplied by Vencorex^®^ Chemicals (Saint Priest, France) and used as received. Velvetol^®^ H500 was kindly supplied by WeylChem International GmbH (Frankfurt am Main, Germany) and used as received.

### 2.2. Methods

Isocyanate equivalent weight (IEW) and hydroxyl equivalent weight (HEW)

The IEW and HEW of LDI and castor oil, respectively, were determined by ^1^H NMR titration, using 1,3,5-trioxane as the internal standard. For each compound, three samples were prepared with approximately 50 mg of LDI or castor oil and 20 mg of 1,3,5-trioxane, dissolved in 0.5 mL of CDCl_3_ and analyzed by ^1^H NMR. The IEW and HEW were determined by integration of the signals at 3.31 ppm and 4.01 ppm for LDI, 3.57 ppm for castor oil, and 5.12 ppm for 1,3,5-trioxane (LDI IEW = 116 g·eq^−1^ and castor oil HEW = 336 g·eq^−1^).

The IEW of Desmodur^®^ eco N 7300 and Tolonate™ X FLO 100 and HEW of Velvetol^®^ H500 were determined from the NCO content and hydroxyl number, respectively, provided by the corresponding certificates of analysis (Desmodur^®^ eco N 7300: 22.0%, IEW = 173 g·eq^−1^; Tolonate™ X FLO 100: 12.40%. IEW = 341 g·eq^−1^; Velvetol^®^ H500: 218.1 mg KOH·g^−1^, HEW = 257 g·eq^−1^). The HEW values of chain extenders (glycerol and 1,3-propanediol) were calculated from the molar masses and functionalities (glycerol HEW = 31 g·eq^−1^ and 1,3-propanediol HEW = 39 g·eq^−1^).

#### 2.2.1. Nuclear Magnetic Resonance (NMR)

The nuclear magnetic resonance (NMR) spectra were recorded on a Bruker Avance™ III 400 MHz spectrometer. The instrumental parameters for recording ^1^H NMR spectra were as follows: flip angle = 30°; acquisition time = 4 s; pulse delay = 1 s; number of scans = 16; pulse width = 3.08 μs.

#### 2.2.2. Fourier Transform Infrared (FTIR)

The Fourier transform infrared (FTIR) spectra were recorded using attenuated total reflection (ATR) in transmission mode with a ThermoScientific Nicolet iS50 FT-IR Flex Gold spectrometer equipped with a deuterated triglycine sulfate (DTGS) detector. The characteristic IR absorption bands are reported in cm^−1^.

#### 2.2.3. Thermogravimetric Analyses (TGA)

Thermogravimetric analyses (TGA) of the cured polyurethanes were performed on a Netzsch STA 449 F1 TGA under 50 mL·min^−1^ argon or air flow. The protective gas used was argon with a 20 mL·min^−1^ flow. Approximately 10–20 mg of sample was placed in an alumina crucible and heated from room temperature to 800 °C with a 20 °C·min^−1^ heating ramp.

#### 2.2.4. Differential Scanning Calorimetry (DSC)

Differential scanning calorimetry (DSC) analyses were carried out using a NETZSCH DSC200F3 calorimeter. Constant calibration was performed using indium, *n*-octadecane, and *n*-octane standards. Nitrogen was used as the purge gas at 40 mL·min^−1^. Approximately 10–20 mg of sample was placed in pierced aluminum pans and the thermal properties were recorded between −150 and 200 °C at 20 °C·min^−1^ to observe the glass transition temperature. The glass transition temperatures (*T_g_*) were measured on the second heating ramp in order to erase the thermal history of the polymer. All of the reported temperatures are middle values.

#### 2.2.5. Dynamic Mechanical Analyses (DMA)

Dynamic mechanical analyses (DMA) were carried out on a Metravib DMA 25 instrument with Dynatest 6.8 software. Uniaxial stretching of samples was performed while heating at a rate of 3 °C·min^−1^ from −110 to 100 °C, keeping the frequency at 1 Hz with a fixed strain (10^−5^ m).

#### 2.2.6. Cross-Linking Density

The crosslinking density was determined according to the theory of rubber elasticity for small deformations on the rubbery plateau [64,65]. The crosslink density *ν* was obtained from Equation (1), with *E*′ being the storage modulus, *R* being the molar gas constant, and *T_α_* being the temperature of the α transition at the maximum of the *tan δ* curve.

(1) $$\upsilon \left(mol\cdot m^{-3}\right)=\frac{E\prime_{T_{\alpha }+50}}{3\;R(T_{\alpha }+50)}$$

#### 2.2.7. Gelation Times

Gelation times were determined using a ThermoFischer Mars 60 instrument using a plate–plate aluminum disposable geometry (25 mm diameter, 0.4 mm gap). Measurements were performed using a multi-frequency program at 80 °C. Approximatively 0.1 mL of the formulation was used for each measurement and the acquisition process was started after 90 s of stabilization at the measurement temperature. The multi-frequency program allowed the measurements to be performed at 1, 2.6, and 7 Hz within the same experiment. A stress of 3 Pa was used and distributed proportionally.

#### 2.2.8. Swelling Index

Three samples of around 50 mg each were separately immersed in THF for 24 h. The swelling index (*SI*) was calculated using Equation (2), where m_1_ is the mass of the material after swelling in THF and m_2_ is the initial mass of the material.

(2) $$SI\;\left(\%\right)=\frac{m_{1}-m_{2}}{m_{2}}\times 100$$

#### 2.2.9. Gel Content

After SI measurements, the samples were dried in a ventilated oven at 70 °C for 24 h. The gel content (*GC*) was calculated using Equation (3), where m_3_ is the mass of the material after the oven and m_2_ is the initial mass of the material.

(3) $$GC\;\left(\%\right)=\frac{m_{3}}{m_{2}}\times 100$$

#### 2.2.10. Shore Hardness A

The Shore hardness A of the materials was measured at room temperature using a Sauter HDD 100-1 Shore A durometer. The maximum value of this durometer is 100 ShA, with a precision of 0.1 ShA. The values are denoted as the means of 3 successive measures with the associated standard deviation.

#### 2.2.11. Synthesis of PUs with Castor Oil (One-Pot Method)

As a representative example, 2.57 g of LDI (IEW = 116 g·eq^−1^) and 7.43 g of castor oil (HEW = 336 g·eq^−1^) were introduced in a polypropylene (PP) flask. The mixture was mixed with a SpeedMixer™ at 2500 rpm for 2 min and then was transferred into an aluminum pan and introduced in the oven at 80 °C for 24 h. Reagent amounts of materials synthesized by one-pot method are displayed in Table S1 (in Supplementary Materials).

#### 2.2.12. Synthesis of PUs with Velvetol^®^ H500 and a Chain Extender (Two-Step Method)

As a representative example, 5.30 g of LDI (IEW = 116 g·eq^−1^) was introduced in a 50 mL, two-neck, round-bottom flask. The system was purged with nitrogen for 10 min and then heated up to 80 °C. Then, 4.70 g of Velvetol^®^ H500 (HEW = 257 g·eq^−1^) was added with a syringe driver for one hour (2.5 mL·h^−1^) with magnetic stirring. The mixture was stirred for an additional 3 h. Then, the prepolymer (IEW = 482 g·eq^−1^) was transferred to a PP flask and 0.75 g of glycerol (HEW = 31 g·eq^−1^) was added. The formulation was then mixed at 2500 rpm for 2 min in a PP flask with a SpeedMixer™, poured into an aluminum pan, and cured at 80 °C for 24 h in an oven. Reagent amounts of materials synthesized by two-step method are displayed in Table S2 (in Supplementary Materials).

#### 2.2.13. Synthesis of PUs with Velvetol^®^ H500 (One-Pot Method)

As a representative example, 3.20 g of LDI (IEW = 116 g·eq^−1^), 6.46 g of Velvetol^®^ H500 (HEW = 257 g·eq^−1^), and 0.34 g of glycerol (HEW = 31 g·eq^−1^) were introduced in a polypropylene (PP) flask. The mixture was mixed with a SpeedMixer™ at 2500 rpm for 2 min and was then transferred into an aluminum pan and introduced in the oven at 80 °C for 24 h. Reagent amounts of materials synthesized by one-pot method are displayed in Table S3 (in Supplementary Materials).

## 3. Results and Discussion

### 3.1. Synthesis of PUs

**Scheme 1:**
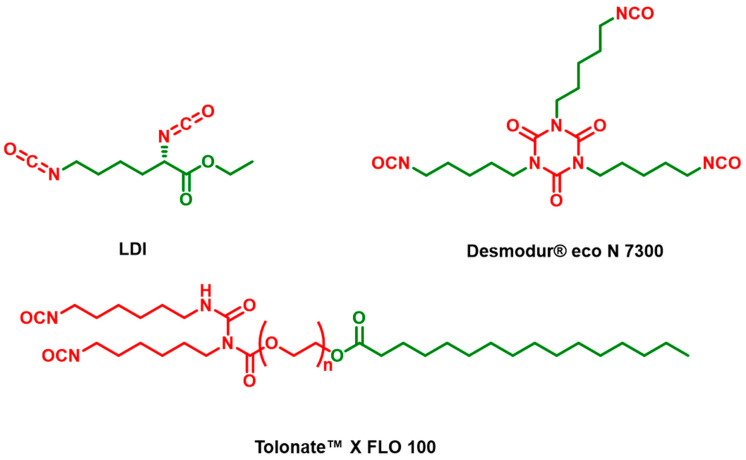
Chemical structures of the commercial partially bio-based isocyanates. Green-colored moieties come from bio-based molecules and red-colored moiety atoms come from petro-sourced molecules.

As mentioned above, three different bio-based polyisocyanates were chosen for this study (Scheme 1). The first one, L-Lysine ethyl ester diisocyanate (LDI), is a diisocyanate synthesized from the ethyl ester of the essential L-lysine amino acid. Since esterification of L-lysine can be carried out with bio-based ethanol, the bio-based carbon content of this monomer can reach 80%. The second one is the Tolonate™ X FLO 100 from Vencorex^®^ Chemicals, a partially bio-based hexamethylene diisocyanate (HDI) allophanate containing 32% bio-based carbons according to its technical datasheet (TDS). The exact chemical formula of this diisocyanate is not disclosed by the manufacturer. However, the electrospray ionization mass spectrometry (ESI-MS negative) image of Tolonate™ X FLO 100, displayed in Figure S1 (in Supplementary Materials), shows several intense signals between 567.38 Da and 1317.90 Da, separated by 44.03 Da. This separation matches with an ethylene glycol unit (·CH_2_-CH_2_-O·), and thus we can conclude that the side-chain of the allophanate contains poly(ethylene glycol). This is also confirmed by ^1^H NMR, where we can observe the characteristic peak of a poly(ethylene glycol) chain at 3.63 ppm in the ^1^H NMR spectrum of Tolonate™ X FLO 100 (Figure S2 in Supplementary Materials). Additionally, Tolonate™ X FLO 100 is synthesized from palm oil, as mentioned previously [47,48] Thus, the side-chain of allophanate should be composed of non-reactive poly(ethylene glycol) end-functionalized by palmitic acid, as depicted in Scheme 1. This hypothesis matches perfectly with the signals between 567.38 Da and 1317.90 Da of the ESI-MS spectra. Thus, given the bio-based content of Tolonate™ X FLO 100 and the proposed chemical structure of this allophanate, we can conclude that the bio-based part is, therefore, the palmitic acid moiety, whereas poly(ethylene glycol) and the chains containing the pendant isocyanate functions probably originate from petro-sourced ethylene glycol and hexamethylene diisocyanate (HDI), respectively. Finally, Desmodur^®^ eco N 7300 from Covestro^®^ is the isocyanurate of pentamethylene-diisocianate (PDI), containing 68% bio-based carbons according to the TDS.

**Scheme 2:**
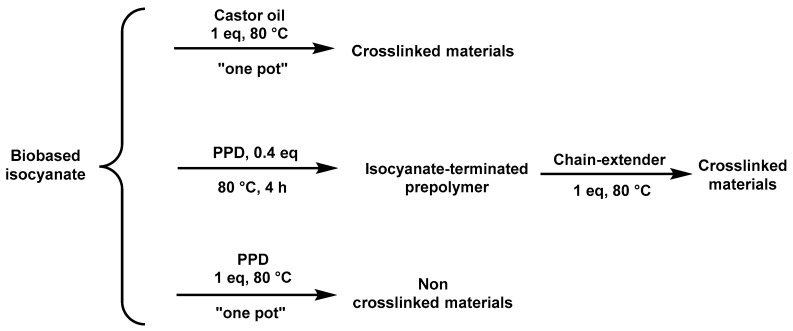
Synthetic pathways for the obtention of polyurethanes from monomers disclosed in Table 1.

**Scheme 3:**
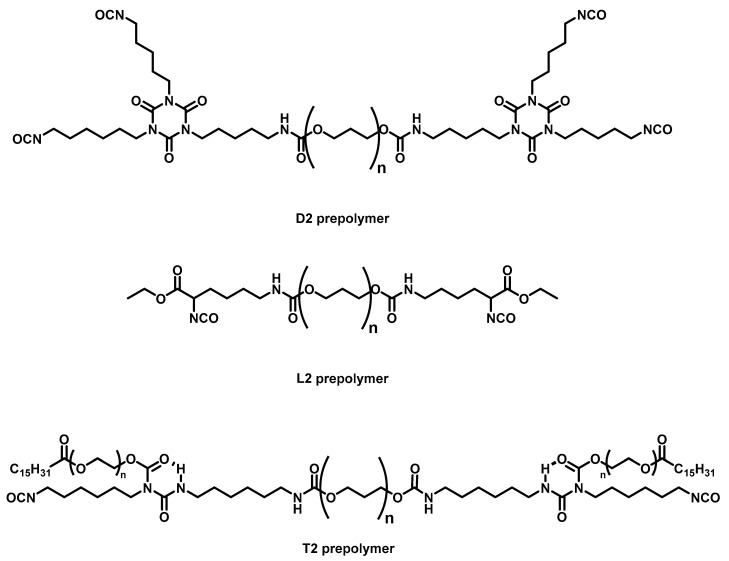
Idealized structures of prepolymers.

Nine different partially bio-based polyurethanes were synthesized by two different methods with these polyisocyanates leading to materials with high bio-based content (from 50% for entry T2 to 95% to entry L1), as displayed in Table 1 and Scheme 2. The first method was a one-pot method where the three different commercial polyisocyanates were reacted with castor oil (L1, T1, D1) or with PPD (L3, T3, D3) with the addition of glycerol for difunctional isocyanate-containing formulations (L3 and T3). Castor oil is mainly composed of the triglyceride of ricinoleic acid, which naturally bears a hydroxyl group on the fatty acid chain. With a mean functionality of 2.7, the different isocyanates used could lead to crosslinked materials. Thus, castor oil was simply mixed with isocyanates in stoichiometric equivalent proportions and the formulations were cured at 80 °C. On the other hand, the second method performed was a two-step method, which consisted of the formation of an isocyanate terminated prepolymer (Scheme 3) by slowly adding the polyol in an excess of isocyanate. Velvetol^®^ H500, which is a difunctional hydroxyl-terminated polypropanediol with an approximate molecular weight of 500 g·mol^−1^, was reacted with each isocyanate in excess at 80 °C in order to form the prepolymer. The addition of the polyether was performed dropwise over 1 h in the excess isocyanate (2.5 mol equivalent, considering that the molar mass of each monomer is the product of the IEW, or HEW for Velvetol^®^ H500, with the theoretical functionality). Then, after the complete addition of the polyol, all the formulations were stirred for an additional 3 h. Then, before curing in an oven, the chain-extender was added to reach the full crosslinking of the prepolymer. The desmodur-based formulation was cured with the addition of a stoichiometric amount of 1,3-propanediol, which has a functionality of 2 and led to the D2 formulation. As Tolonate™ X FLO 100 and the LDI-based prepolymer are difunctional prepolymers, the trifunctional glycerol was used to achieve crosslinking, leading to T2 and L2 materials, respectively. The amount of chain-extender was calculated according to the theoretical functionality obtained for the prepolymers (Table S2), without any additional measurement of the corresponding IEW. All materials were cured for 24 h at 80 °C in order to ensure the complete reaction of the formulations. DSC images showed neither any residual enthalpy, nor any difference of the measured *T_g_* during the first or the second heating ramp, confirming the completion of the reaction. Finally, three polyurethanes (entries L3, T3, and D3) were synthesized by one-pot method using a mixture of Velvetol^®^ H500 (95% *w*/*w*) and glycerol (5% *w*/*w*), except for entry D3, since Desmodur^®^ eco N 7300 is a trifunctional isocyanurate.

Conversion of the prepolymer formations (entries L2, T2, and D2) was monitored by NMR ^1^H spectroscopy (Figures S3–S5 (in Supplementary Materials), respectively), however full conversion of the terminal hydroxyls was not attained in all cases due to the high viscosity of the bulk mixture, which could not be properly mixed with magnetic stirring. The NMR ^1^H spectra of the three synthesized prepolymers showed in all cases the almost complete disappearance of the two signals from the two CH_2_ close to the hydroxyl groups of Velvetol^®^ H500 at 3.71 ppm (t) and 3.57 ppm (t). However, small peaks were still present after 3 h at 80 °C, especially for entry D2, probably because of the high viscosity of the mixture. NMR spectra of entry L2 (Figure S3) exhibited the formation of two new signals centered at 3.33 ppm (t) and at 4.03 ppm (dd), corresponding to the CH and CH_2_, respectively, next to the formed urethane functions. Note that LDI possesses two isocyanates functions in α and ε positions, presumably with different reactivities. In fact, the isocyanate group in the α position should be less reactive than the one in the ε position, since it is a secondary isocyanate. This is supported by the integration values of NMR spectra of LDI and Velvetol^®^ H500 (Figure S3) reactions, whereby the peak at 3.33 ppm (t), corresponding to the CH_2_ in ε-position, decreases faster than the peak α-CH_2_ at 4.03 (dd). However, due to the presence of other peaks partially overlapping with these peaks, it is not possible to accurately quantify the reactivity ratio of the primary and secondary isocyanates in ε and α positions. New signals are clearly visible between 4.10 and 4.25 ppm (m), corresponding to the CH_2_ and α-CH close to the urethane functions of the Velvetol^®^ H500 and LDI, respectively. Moreover, a new peak centered at 3.15 (q) ppm corresponding to the CH_2_ of the LDI moiety next to the urethane group in the ε-position is visible in the spectrum. On the other hand, NMR spectra of entry T2 (Figure S4) also exhibit the peaks centered at 4.11 and 3.15 ppm, corresponding to the CH_2_ of the Velvetol^®^ H500 and Tolonate X FLO 100 moieties, respectively, next to the formed urethanes groups that are also present. In the case of entry D2 (Figure S5), these signals are centered at 4.10 ppm (q) and 3.14 (t) ppm and also correspond to the CH_2_ in the α-position of the urethane linkage of the Velvetol^®^ H500 and Desmodur^®^ eco N 7300 moieties, respectively. Additionally, new signals at 1.85 and 1.53 ppm can also be also observed, corresponding to the CH_2_ in the β-position of the urethane linkages of the Velvetol^®^ H500 and Desmodur^®^ eco N 7300 moieties, respectively. Additionally, the signal at 3.31 ppm (t), corresponding to the CH_2_ next to the isocyanate group, is decreased compared to the peak at 3.90 ppm, which is produced by the CH_2_ next to the isocyanurate group.

### 3.2. Curing Behavior

**Figure 1:**
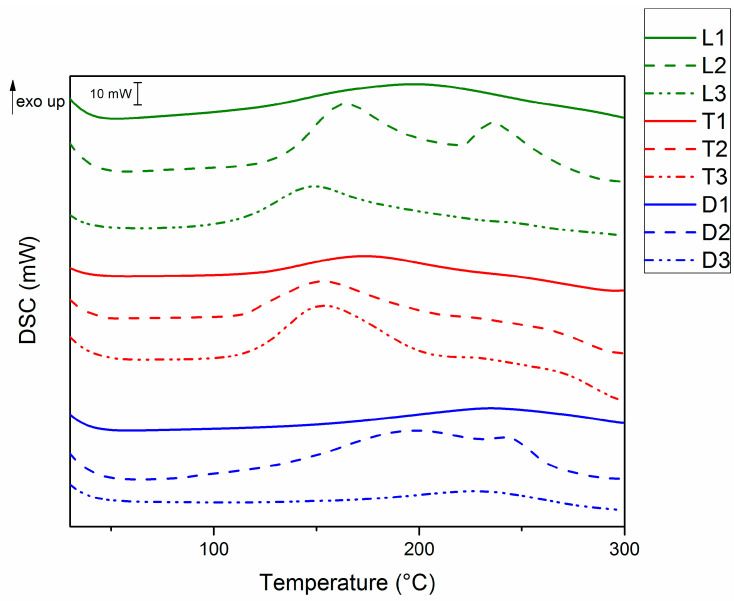
DSC images of uncured formulations at 40 °C·min^−1.^

The curing reaction was monitored by non-isothermal DSC at 40 °C·min^−1^ in order to observe curing exotherms. The low exotherms of the reaction between hydroxyls and isocyanates were difficult to observe with classical heating ramps of 10 or 20 °C·min^−1^, thus we used a more rapid ramp, which amplified the exothermic phenomena. Exothermic peaks were, thus, observed but enlarged on a wider range of temperatures. However, as it can be observed in Figure 1, the curing exotherms of D1, T1, and L1 only show one exothermic phenomenon, which illustrates the presence of only secondary alcohols in castor oil. The reaction of prepolymers with the chain extenders, however, showed different trends. The thermogram of L2 has two distinct peaks, which are slightly overlapped, with peak maxima at 165 and 235 °C. The thermogram of D2 also shows a broad peak starting from 100 °C with a maximum at 200 °C and a shoulder with a maximum at 243 °C. It is likely that these observed peaks correspond to allophanate formation [66,67]. It is known that isocyanate may react and form allophanate moieties at high temperatures, sometimes regardless of the NCO:OH ratio. In our case, the reaction of the prepolymer with the chain extender occurred at lower temperatures than in the castor oil formulations. Thus, crosslinking may have occurred before the allophanate formation in the case of the prepolymers, as shown by the distinct peaks appearing in the thermograms. In addition, we did not perform any measurement of the IEW of the prepolymers and used the theoretical values of IEW. This could lead to non-stoichiometric formulations and a possible excess of isocyanates. However, the second peak was not observed for the T2 formulation. This could simply be explained by the early degradation of the network, as evidenced by the baseline change in the thermogram, and the calculated *T_d_*_5%_ of 290 °C, as assessed by TGA. Finally, the one-pot formulations with Velvetol^®^ H500 all displayed unique reaction exotherms. For LDI, a single peak was observed around 150 °C, slightly lower than the prepolymerized version. Tolonate™ X FLO 100 displayed very similar thermograms between one-pot and prepolymer versions, with a single exotherm, and a baseline derivation at higher temperatures. Finally, the isocyanurate–velvetol formulation without prepolymerization displayed a very low exotherm, similar to the castor oil-based formulation. However, it is important to note that even though the one-pot formulations using Velvetol^®^ H500 displayed similar reactivities than prepolymers or castor-oil formulations, the isothermal curing performed at 80 °C in an oven in order to obtain thermosetting materials was not successful. All of the one-pot formulations with Velvetol^®^ H500 led to viscous liquids or mixtures of solid and liquid phases. DSC performed after the curing at 80 °C did not show any residual enthalpy, which demonstrates the completion of the reaction. The obtention of non-crosslinked materials, despite the disappearance of any reaction exotherm, can be explained by a possible immiscibility of Velvetol^®^ H500 and isocyanates, leading to a non-homogeneous mixture, which does not statistically lead to infinite macromolecules. DSC images after curing at 80 °C for 24 h did not show any residual enthalpy, as shown in Figure S6 (in Supplementary Materials). This reaction behavior led us to use Velvetol^®^ H500 only as a prepolymer precursor, while L3, T3, and D3 formulations were not further investigated.

In order to fully compare the curing behavior, DSC images are not sufficient, since the assessment of curing kinetics by DSC should be performed using several heating ramps, with the limitations described above [68]. Nevertheless, observation of gelation times following an isothermal heating program can be meaningful for the comparison of the reactivity of different formulations [69]. Thus, multi-frequency gelation time determination was performed on a rheometer using plate–plate geometry at 80 °C. A stress of 3 Pa was applied proportionally between the three frequencies. Gelation times were determined for each frequency as the time for which *G*’= *G*”, while Table 2 shows gelation time values obtained at the three measured frequencies and the average gelation times. *G*’ and *G*” curves are also displayed in Figures S7–S12 (in Supplementary Materials). Tolonate™ X FLO 100 and PDI isocyanurate formulations showed the same trend, reaching gelation faster when used as PPD prepolymers. This trend could have been expected, since the addition of a small molecular weight chain extender in prepolymers would have led to a fast-growing network, and thus a shorter time to obtain a gel. This trend was observed regardless of the functionality of the prepolymer, e.g., 2 for HDI allophanate and >2 for PDI isocyanurate. HDI allophanate–castor oil attained gelation after 5 h on average, probably due to the side chain of Tolonate, which could slower the reaction by hindering the network and preventing the collision between reactive species. This hindering may not happen with Tolonate prepolymer, due to a more favorable chain conformation, especially since one may consider that POE and PPD blocks have similar interactions. On another hand, LDI formulations showed a different trend, with the highest gelation time obtained for the prepolymer. This trend was unexpected since the non-isothermal DSC displayed the narrowest reaction exotherms, and thus fastest reaction rates. However, it shall be noted that rheology measurements have been performed in isothermal conditions and at a relatively moderate temperature (80 °C). Considering the difference of reactivity between the two isocyanates moieties (i.e., NCO attached to CH_2_ vs. NCO attached to CH), as evidenced by the NMR ^1^H monitoring of prepolymerization reaction (also performed at 80 °C), a lower reactivity of isocyanates end chain may be observed in that case. Indeed, if the NCO attached to CH_2_ reacts first in prepolymerization, end-chain NCO of the prepolymer are mainly attached to the CH of LDI, thus displaying a lower reactivity. Even in one-step formulations, LDI formulation displayed the lowest reactivity, thus the results are consistent. In overall, in one-step formulations, the reactivity of isocyanates follows the trend PDI isocyanurate > LDI > HDI allophanate, whereas in prepolymer, the trend is PDI isocyanurate < HDI allophanate < LDI. It is noted that in all cases, the isocyanurate was the most reactive, but it has also the highest isocyanate functionality among the studied isocyanates.

FTIR (ATR) spectra of cured materials made with castor oil by one-pot method (entries L1, T1, and D1) or made with a prepolymer and a chain extender (two-step method) are displayed in Figures S13 and S14 (in Supplementary Materials), respectively. In all cases, a broad band between 3600 and 3200 cm^−1^ is present in the spectra of the six different materials, corresponding to the N–H bond of the urethane groups formed during the curing process. Moreover, the absence of a band at 2300–2200 cm^−1^ characteristic of isocyanates groups indicates that the curing reaction was completed in all cases, except for D1, where a small signal is observed at 2280 cm^−1^, suggesting the presence of some residual isocyanate functions. A very strong peak is also present in all cases at 1700 cm^−1^. Note that this band for materials possessing isocyanurates (entries D1 and D2) is visible as a shoulder of a stronger band at 1670 cm^−1^ produced by isocyanurates. In addition, these entries exhibited the carbonyl band at slightly lower wavenumbers than T1, T2, L1, and L2, probably because of the hydrogen bond between the urethane linkages. Indeed, the isocyanurate structure involved a higher urethane bond density in entries D1 and D2, increasing the hydrogen-bonded urethanes groups and then the crosslinking density. On the other hand, the bands of esters of LDI and Tolonate™ X FLO 100 (entries L1, L2, T1 and T2) are probably overlapped by the peak of urethane at 1700 cm^−1^. Additionally, these entries showed another strong band at 1640 cm^−1^ also produced by the isocyanurate groups. FTIR was also performed on the one-pot formulations using Velvetol^®^ H500 after the curing at 80 °C, as shown in Figure S15 (in Supplementary Materials). As confirmed by DSC, the isocyanate reaction was complete after 24 h, since no residual signal for isocyanate was observed.

### 3.3. Thermomechanical Properties

**Figure 2:**
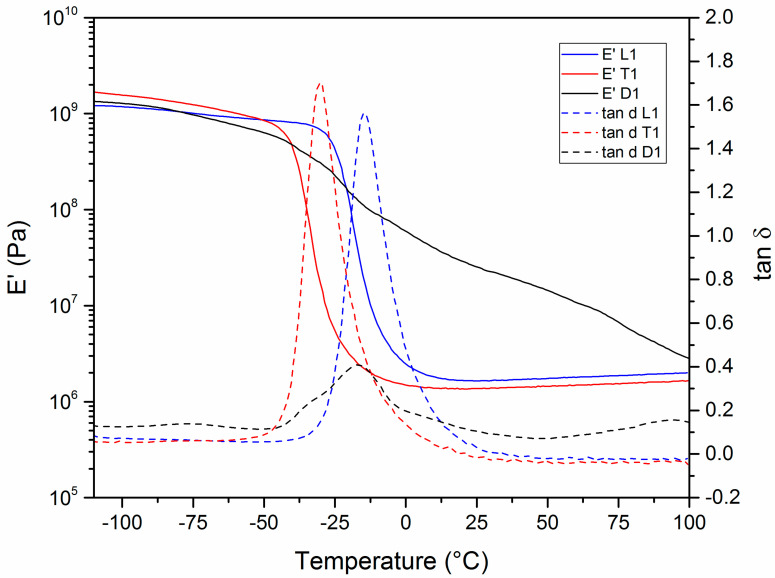
Storage modulus (E’) and tan δ vs. temperature values for cured materials for entries L1, T1, and D1, obtained using DMA.

Viscoelastic behavior of the six cured materials synthesized in this work have been studied by DMA and the results are displayed in Table 3. Glassy and rubbery storage moduli have been measured for all the samples at *T_α_* − 50 °C and *T_α_* + 50 °C, respectively. The alpha-transition temperatures (*T_α_*) have been measured at the maximum of the tan δ for each entry. Thermograms of entries L1, T1 and D1 representing the storage modulus (*E’*) and *tan δ* as function of the temperature are displayed in Figure 2. Entries L1, T1, and D1, synthesized by one-pot method with castor oil, exhibited *T_α_* transitions at −14 °C, −30 °C, and –17 °C, respectively. The storage moduli of the three materials in the glassy region were rather similar and decreased slightly with the increasing temperature, with values at *T_α_* − 50 °C of 0.86 GPa for D1, 0.93 GPa for L1, and 1.9 GPa for T1. After the alpha transition, entries L1 and T1 reached a plateau, suggesting that the curing reaction was complete. L1 exhibited a higher value at *T_α_* + 50 °C (1.7 MPa), which may have been correlated with a higher crosslinking density, while T1 showed a value of 1.4 MPa, in good agreement with the already reported value [41]. However, entry D1 did not reach a plateau after the alpha transition and decreased continuously with the temperature, suggesting that the material was not completely crosslinked. In fact, *tan δ* values of entry D1 increased somewhat from 75 to 100 °C, suggesting that the material was not completely cured. Additionally, the relatively broad peaks and low magnitude of *tan δ* indicate some heterogeneity of the material structures, especially for D1. The crosslinking densities that were determined on the rubbery plateau showed that L1 and T1 had similar crosslinking densities of 221 and 192 mol·m^−3^, respectively. Both difunctional isocyanates had similar crosslinking architecture, since both polyol and chain extenders are similar. However, T1 had the lowest crosslinking density, since it possesses the highest molecular weight. The value was not determined for D1, since no plateau was observed.

**Figure 3:**
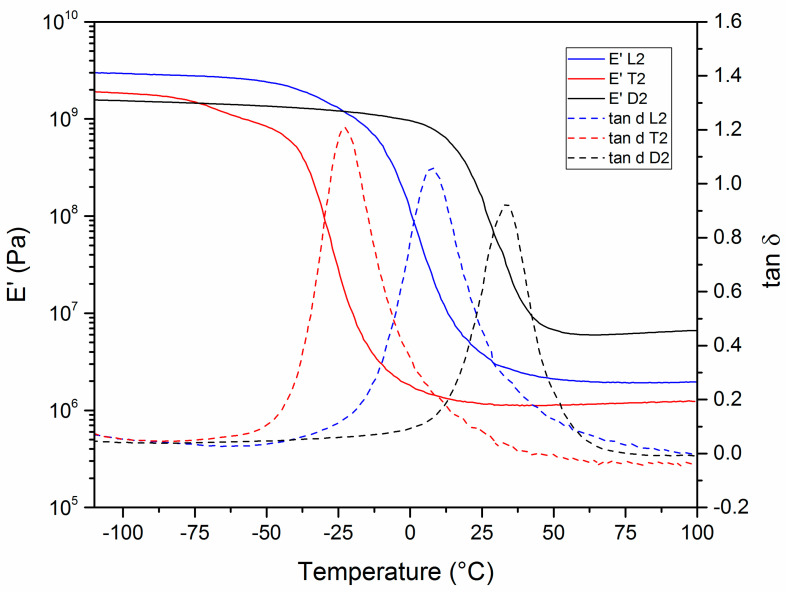
Storage modulus (E’) and tan δ vs. temperature values for cured materials for entries L2, T2, and D2, obtained using DMA.

L2, T2, and D2 thermograms obtained using DMA are displayed in Figure 3 and data are available in Table 3. The three materials synthesized by two-step method presented similar behaviors. T2 exhibited the lowest *T_α_* (−23 °C), probably due to the presence of the fatty acid chains, whereas D2 showed the highest value (+33 °C). Regardless of the material synthesized by two-step method, no decrease of the storage modulus was observed after the alpha transition and the three samples reached a plateau, suggesting that the curing reaction was completed. The measured storage moduli at *T_α_* + 50 °C were 2.0, 1.2, and 6.3 MPa for L2, T2, and D2, respectively. Thus, D2, which exhibited the highest storage modulus in the glassy plateau, possesses the highest crosslink density, followed by L2 and finally T2. Regarding crosslinking densities, T2 and L2 have similar densities, similar to T1 and L1. L2 showed a slightly higher crosslinking density than L1 (243 and 221 mol·m^−3^, respectively), consistent with the higher *T_α_* obtained. The opposite trend was observed for Tolonate, where T2 displayed the lower *ν* (160 mol·m^−3^) compared to T1 (192 mol·m^−3^). This behavior could be due to the side-chain of Tolonate™ X FLO 100, which could act as an internal plasticizer of the network. This may have more impact in the prepolymer route, which entails a higher density of polyether in the network. This is consistent with the marginal impact of the polymerization method on *T_g_* and *T_α_* for Tolonate™ X FLO 100 in our formulations. Finally, as expected, D2 displayed the highest crosslinking density, consistent with *T_α_* and DSC results, as discussed below.

**Figure 4:**
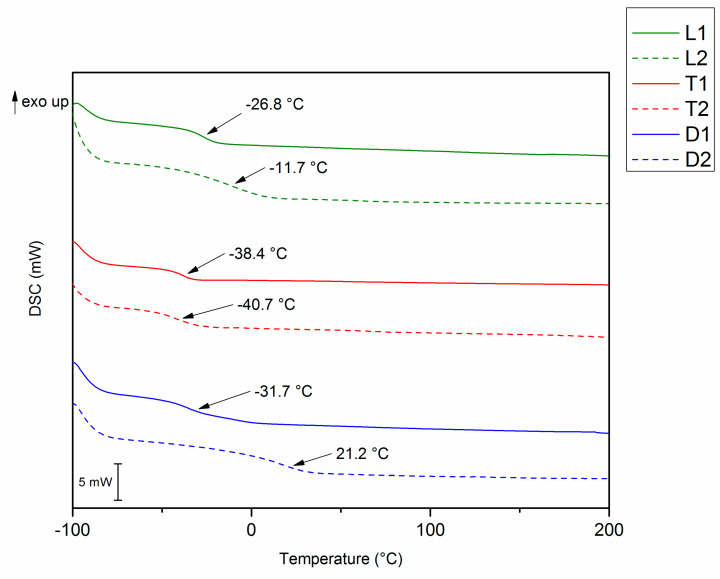
DSC thermograms of cured materials for entries L1, L2, T1, T2, D1, and D2.

**Figure 5:**
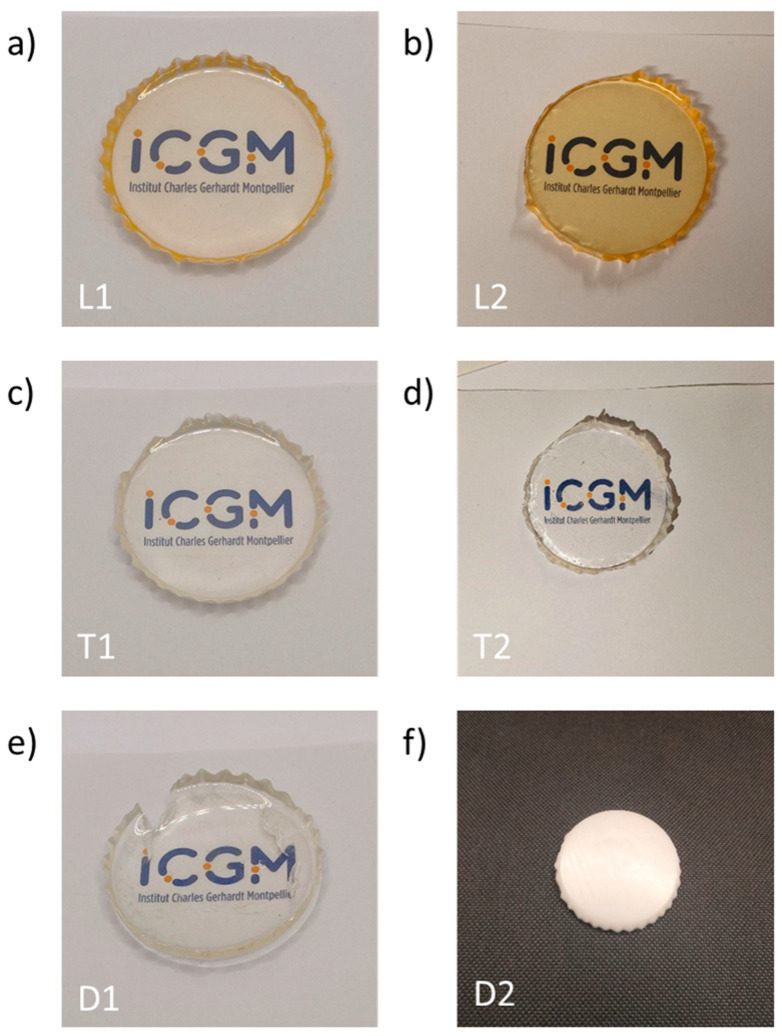
Photographs of cured materials: (**a**) L1; (**b**) L2; (**c**) T1; (**d**) T2; (**e**) D1; (**f**) D2.

Glass transition temperatures for the thermosets were determined after curing at 80 °C overnight for each formulation. The *T_g_* was determined by DSC on the second heating ramp at 20 °C·min^−^^1^ as the temperature at the middle of the inflexion. The results are shown in Figure 4, with plain lines used for the castor-oil-based formulations and with dashed lines for the two-step formulations. As can be noted, the glass transition temperatures are roughly similar to castor oil-based formulations, namely −26.8 °C for L1, −38.4 °C for T1, and −31.7 °C for D1. The macroscopic observation of all these materials revealed amorphous transparent networks, with D1 and T1 being colorless and L1 being slightly yellow, as displayed in Figure 5a,c,e. The glass transition temperatures of the formulations using prepolymers with polypropanediol exhibited different behaviors depending on the functionality of the isocyanate. Indeed, both difunctional isocyanates gave low *T_g_*s values, similar to castor-oil-based formulations, i.e., −11.7 °C for LDI–PPD and −40.7 °C for Tolonate–PPD. However, the isocyanurate-based thermoset displayed a *T_g_* of +21.2 °C, which is higher. The differences in *T_g_* values between castor-oil- and PPD-based thermosets were 15.8 °C in the case of LDI, 2.3 °C in the case of Tolonate, and 52.9 °C in the case of the isocyanurate. It must be noted that when compared with DMA, the *T_g_* observed using DSC was obvious, whereas the *tan δ* peak was weak and broad. This may be explained by the network being non-homogeneous, especially because both isocyanate and polyol are trifunctional and secondary alcohols of castor oil are poorly reactive. Hence, some unreacted moieties may still be present or pendant chains may remain and plasticize the network, with a poorly defined mechanical response. However, the macroscopic observation of the polyurethane material did not evidence such heterogeneity, since it is transparent. It has also to be noted that mixing isocyanurate and castor oil when formulating the polyurethane obviously led to an emulsion and then phase separation. However, besides transparency, the macroscopic examination of cured D1 revealed that it was quite tacky and partly broke during unmolding. On the other hand, the D2 material, which appeared to be very homogeneous with clear transitions in both DMA and DSC, was opaque, revealing a segmented network, with hard clusters that were likely composed of PPD–isocyanurate adducts. The two other crosslinked prepolymers, namely L2 and T2, were amorphous, transparent, and soft. L2 had a pronounced yellow color, whereas T2 was colorless, as shown in Figure 5b,d,f.

The thermal stability of cured networks was assessed using TGA under argon and air conditions, with the results displayed in Figure S16 (in Supplementary Materials) and Table 3. The results are quite similar under both atmospheres, with *T_d_*_5%_ values ranging from 292 to 326 °C for all materials. The degradation pathways seemed to be similar for all crosslinked networks. Previous studies, especially on PPD-based polyurethane, have shown that the polyether part of the polymers degrades at high temperatures of around 400 °C [70]. It has also been shown that in castor-oil-based polyurethanes, degradation of the urethane bonds occurs before the degradation of the polyol part [29,30]. In our work, isocyanurate-based thermosets have the highest degradation temperatures, which may result from the higher stability of the isocyanurate moiety, which confers rigidity and stability to the network. It is noteworthy that networks based on aliphatic isocyanates still displayed relatively high thermal stabilities. For instance, poly(ester-urethanes) based on LDI have shown low thermal stabilities in the literature, limiting the applications to mostly biomedical applications [71]. Among all of the networks investigated, the T2 network displayed the lowest thermal stability. This can be explained by the fatty acid and PEO side-chain, which could correspond to the most fragile part of the network. The fact that it presents a lower thermal stability than the T1 network may also be explained by its lowest *T_g_* and rubbery Young’s modulus. T2 globally has the lowest thermomechanical properties. Our new bio-based formulations could, therefore, broaden the applications of such isocyanates. At 800 °C, no obvious difference was observed between thermograms under either air or argon conditions, and for all samples, no residue was observed at this temperature, except for D2 under argon conditions, which showed a residual mass of up to 4%. The absence of any aromatic moiety explains the absence of residual mass at high temperatures. Several degradation steps were observed, corresponding to the cleavage of the different linkages inside the network, however all samples were fully degraded under argon conditions at 500 °C and at around 600 °C under air conditions.

### 3.4. Swelling Index, Gel Content, and Shore Hardness Values

Swelling index (SI) and gel content (GC) values after 24 h in THF, as well as the Shore hardness A and D conditions for all cured materials, were measured and the results are shown in Table 4. In all cases, materials synthesized by one-pot method with castor oil exhibited higher SI and lower GC values than the ones synthesized by two-step method with the same polyisocyanate. This indicates a lower crosslink density for the materials synthesized with castor oil compared to homologous materials synthesized by two-step method, as already shown by DMA analysis. This phenomenon was especially significant in entries D1 and D2, where the SI values of D1 were almost double those of D2, while the GC of D1 dropped significantly (75% for D1 and 98% for D2). In fact, D2 presented remarkably low SI and high GC values, suggesting that it had a high crosslink density. SI and GC values indicated that the materials synthesized with LDI possess a slightly higher crosslink density than the ones synthesized with Tolonate™ X FLO 100, which exhibited the highest SI (403–420%) and lowest GC (92–94%) values of all synthesized materials except D1, in agreement with DMA analyses.

All of the materials showed similar Shore hardness A values, except for L1, which showed the lowest value at 38.0 ± 0.2, as well as D2, which was the hardest, reaching values of 85.2 ± 1.2 ShA. This can be explained by the *T_g_* value of this latest entry, which was above room temperature.

## 4. Conclusions

Overall, we synthesized six bio-based thermosetting polyurethanes, with *T_g_* values in the range −40 to 21 °C, with very high renewable carbon contents ranging from 50 to 95%. The isocyanurate-based thermoset displayed the highest transition temperatures when used as a prepolymer with polypropanediol, whereas all other materials exhibited negative *T_g_* values. Different reactivities were observed via gelation time determination, with different trends. Th elysine-based isocyanate was the most reactive isocyanate, while HDI allophanate was the most reactive prepolymer. PDI isocyanurate gave the most reactive formulations in all cases but did not achieve full crosslinking with castor oil in stoichiometric proportions. The thermal stabilities of all networks were similar regardless of the TGA atmosphere, with Td5% values of around 300 °C for all materials. These results showed that a wide range of thermoset properties may be obtained with these commercially available monomers and could interestingly improve the sustainability of polyurethanes for various applications.

## Figures and Tables

**Table 1:** Reagents and methods for each formulation and their bio-based carbon contents.

Entry	Polyisocyanate	Polyol	Method (Chain Extender)	Bio-Based Carbon Content (%)
L1	LDI	Castor oil	One-pot	95
L2	LDI	Velvetol^®^ H500	Two-step (glycerol)	90
L3	LDI	Velvetol^®^ H500 + 5 wt% Glycerol	One-pot	94
T1	Tolonate™ X FLO 100	Castor oil	One-pot	66
T2	Tolonate™ X FLO 100	Velvetol^®^ H500	Two-step (glycerol)	50
T3	Tolonate™ X FLO 100	Velvetol^®^ H500 + 5 wt% Glycerol	One-pot	61
D1	Desmodur^®^ eco N 7300	Castor oil	One-pot	89
D2	Desmodur^®^ eco N 7300	Velvetol^®^ H500	Two-step (1,3-propanediol)	79
D3	Desmodur^®^ eco N 7300	Velvetol^®^ H500	One-pot	87

**Table 2:** Gelation times of polyurethanes formulations.

Frequency	Gelation Times (min)					
	L1	L2	T1	T2	D1	D2
1 Hz	140	328	327	80	107	32
2.6 Hz	142	435	332	83	107	35
7 Hz	143	513	340	88	108	38
Average	142 ± 2	425 ± 93	333 ± 7	84 ± 4	107 ± 1	35 ± 3

**Table 3:** Thermomechanical analysis data for cured materials. Note: n.d. = non determined.

Sample	*T_g_* (°C) ^a^	*T_α_* (°C) ^b^	*E*’ (Pa) (Glassy)	*E*’ (Pa) (Rubbery)	*ν* (mol·m^−3^)	*T_d_*_5%_ (°C)	Char Yield (800 °C, %)	*T_d_*_5%_ (°C)	Char Yield (800 °C, %)
			(*T_α_* − 50 °C)	(*T_α_* + 50 °C)		Argon		Air	
L1	−27	−14	9.3·10^8^	1.7·10^6^	221	316	1	317	0
L2	−12	7	2.2·10^9^	2.0·10^6^	243	299	2	288	0
T1	−38	−30	1.3·10^9^	1.4·10^6^	192	303	0	297	0
T2	−41	−23	1.4·10^9^	1.2·10^6^	160	292	0	290	0
D1	−32	−17	8.6·10^8^	2.1·10^7^	n.d.	326	0	332	0
D2	21	33	1.1·10^9^	6.3·10^6^	710	315	4	319	1

Note: ^a^ measured by DSC; ^b^ measured by DMA.

**Table 4:** Swelling index (SI) and gel content (GC) values in THF (24 h) and Shore hardness A values of cured materials.

Entry	SI (%)	GC (%)	Shore A Hardness (ShA)
L1	353	94	38.0 ± 0.2
L2	266	96	57.1 ± 3.0
T1	403	92	49.4 ± 0.4
T2	420	94	41.2 ± 1.0
D1	275	75	57.3 ± 1.0
D2	107	98	85.2 ± 1.2

## Data Availability

Not applicable.

## Supplementary Materials

The following are available online at https://www.mdpi.com/article/10.3390/polym13081255/s1: Table S1: Experimental polyisocyanate and castor oil amounts of entries L1, T1, and D1. Table S2: Experimental polyisocyanate, Velvetol^®^ H500, and chain extender amounts of entries L2, T2, and D2. Table S3: Experimental polyisocyanate, Velvetol^®^ H500, and glycerol amounts of entries L3, T3, and D3. Figure S1: ESI-MS spectra (negative) of Tolonate X FLO 100 in methanol. Figure S2: ^1^H NMR spectrum (400 MHz, CDCl_3_) of Tolonate™ X FLO 100. Figure S3: ^1^H NMR spectra (400 MHz, CDCl_3_) of LDI and Velvetol^®^ H500 reactions at 0, 1, 2, and 3 h after the complete addition of Velvetol^®^ H500. Figure S4: ^1^H NMR spectra (400 MHz, CDCl_3_) of Tolonate™ X FLO 100 and Velvetol^®^ H500 reactions at 0, 1, 2, and 3 h after the complete addition of Velvetol^®^ H500. Figure S5: ^1^H NMR spectra (400 MHz, CDCl_3_) of Desmodur^®^ eco N 7300 and Velvetol^®^ H500 reactions at 0, 1, 2, and 3 h after the complete addition of Velvetol^®^ H500. Figure S6: DSC imagery of one-pot formulations L3, T3, and D3 with Velvetol H500 at 40 °C·min^−1^ after curing at 80 °C for 24 h. Figure S7: Gelation time determination for L1 at 80 °C. Figure S8: Gelation time determination for L2 at 80 °C. Figure S9: Gelation time determination for T1 at 80 °C. Figure S10: Gelation time determination for T2 at 80 °C. Figure S11: Gelation time determination for D1 at 80 °C. Figure S12: Gelation time determination for D2 at 80 °C. Figure S13: FTIR spectra (ATR) of cured materials of entries L1, T1, and D1. Figure S14: FTIR spectra (ATR) of cured materials of entries L2, T2, and D2. Figure S15: FTIR spectra (ATR) of cured materials of entries L3, T3, and D3. Figure S16: TGA (20 °C·min^−1^) results for crosslinked polyurethane formulations: (**a**) one-step formulations under Argon conditions; (**b**) one-step formulations under air conditions; (**c**) two-steps formulations under Argon conditions; (**d**) two-step formulations under air conditions.

## References

[1] Skoczinski P., Chinthapalli R., Carus M., Baltus W., Guzman d.D., Käb H., Raschka A., Ravenstijn J. (2019). Bio-Based Building Blocks and Polymers-Global Capacities, Production and Trends 2019–2024.

[2] Álvarez-Chávez C.R., Edwards S., Moure-Eraso R., Geiser K. (2012). Sustainability of bio-based plastics: General comparative analysis and recommendations for improvement. J. Clean. Prod..

[3] Caillol S., Boutevin B., Pascault J.-P. (2017). Bio-Sourced Epoxy Monomers and Polymers. Handbook of Adhesive Technology.

[4] Nikafshar S., Zabihi O., Hamidi S., Moradi Y., Barzegar S., Ahmadi M., Naebe M. (2017). A renewable bio-based epoxy resin with improved mechanical performance that can compete with DGEBA. RSC Adv..

[5] Rad E.R., Vahabi H., de Anda A.R., Saeb M.R., Thomas S. (2019). Bio-epoxy resins with inherent flame retardancy. Prog. Org. Coat..

[6] Vahabi H., Rohani Rad E., Parpaite T., Langlois V., Saeb M.R. (2019). Biodegradable polyester thin films and coatings in the line of fire: The time of polyhydroxyalkanoate (PHA)?. Prog. Org. Coat..

[7] Winnacker M., Rieger B. (2016). Biobased Polyamides: Recent Advances in Basic and Applied Research. Macromol. Rapid Commun..

[8] Bayer O., Siefken W., Rinke H., Orthner L., Schild H. (1937). A Process for the Production of Polyurethanes and Polyureas [Verfahren zur Herstellung von Polyurethanen bzw. Polyharnstoffen].

[9] Akindoyo J.O., Beg M.D.H., Ghazali S., Islam M.R., Jeyaratnam N., Yuvaraj A.R. (2016). Polyurethane types, synthesis and applications—A review. RSC Adv..

[10] Rokicki G., Parzuchowski P.G., Mazurek M.M. (2015). Non-isocyanate polyurethanes: Synthesis, properties, and applications. Polym. Adv. Technol..

[11] Cornille A., Auvergne R., Figovsky O., Boutevin B., Caillol S. (2017). A perspective approach to sustainable routes for non-isocyanate polyurethanes. Eur. Polym. J..

[12] Delebecq E., Pascault J.-P., Boutevin B., Ganachaud F. (2013). On the Versatility of Urethane/Urea Bonds: Reversibility, Blocked Isocyanate, and Non-isocyanate Polyurethane. Chem. Rev..

[13] Kathalewar M.S., Joshi P.B., Sabnis A.S., Malshe V.C. (2013). Non-isocyanate polyurethanes: From chemistry to applications. RSC Adv..

[14] Maisonneuve L., Lamarzelle O., Rix E., Grau E., Cramail H. (2015). Isocyanate-Free Routes to Polyurethanes and Poly(hydroxy Urethane)s. Chem. Rev..

[15] Błażek K., Datta J. (2019). Renewable natural resources as green alternative substrates to obtain bio-based non-isocyanate polyurethanes-review. Crit. Rev. Environ. Sci. Technol..

[16] Quienne B., Poli R., Pinaud J., Caillol S. (2021). Enhanced aminolysis of cyclic carbonates by β-hydroxylamines for the production of fully biobased polyhydroxyurethanes. Green Chem..

[17] Yebo L., Xiaolan L., Shengjun H. (2015). Bio-Based Polyols and Polyurethanes.

[18] Hojabri L., Kong X., Narine S.S. (2010). Novel long chain unsaturated diisocyanate from fatty acid: Synthesis, characterization, and application in bio-based polyurethane. J. Polym. Sci. Part. A Polym. Chem..

[19] Kyriacos D. (2020). Biobased Polyols for Industrial Polymers.

[20] Dworakowska S., Bogdal D., Prociak A. (2012). Microwave-Assisted Synthesis of Polyols from Rapeseed Oil and Properties of Flexible Polyurethane Foams. Polymers.

[21] Lligadas G., Ronda J.C., Galià M., Cádiz V. (2010). Plant Oils as Platform Chemicals for Polyurethane Synthesis: Current State-of-the-Art. Biomacromolecules.

[22] Seydibeyoğlu M.O., Misra M., Mohanty A., Blaker J.J., Lee K.-Y., Bismarck A., Kazemizadeh M. (2012). Green polyurethane nanocomposites from soy polyol and bacterial cellulose. J. Mater. Sci..

[23] Tenorio-Alfonso A., Sánchez M.C., Franco J.M. (2020). A Review of the Sustainable Approaches in the Production of Bio-based Polyurethanes and Their Applications in the Adhesive Field. J. Polym. Environ..

[24] Furtwengler P., Avérous L. (2018). Renewable polyols for advanced polyurethane foams from diverse biomass resources. Polym. Chem..

[25] Trovati G., Suman M.V.N., Sanches E.A., Campelo P.H., Neto R.B., Neto S.C., Trovati L.R. (2019). Production and characterization of polyurethane castor oil (Ricinus communis) foam for nautical fender. Polym. Test..

[26] Rwahwire S., Tomkova B., Periyasamy A.P., Kale B.M., Koronis G., Silva A. (2019). Chapter 3—Green thermoset reinforced biocomposites. Green Composites for Automotive Applications.

[27] Severino L.S., Auld D.L., Baldanzi M., Cândido M.J.D., Chen G., Crosby W.L., Tan D., He X., Lakshmamma P., Lavanya C. (2012). A Review on the Challenges for Increased Production of Castor. Agron. J..

[28] Pfister D.P., Xia Y., LaRock R.C. (2011). Recent Advances in Vegetable Oil-Based Polyurethanes. ChemSusChem.

[29] Ghasemlou M., Daver F., Ivanova E.P., Adhikari B. (2019). Polyurethanes from seed oil-based polyols: A review of synthesis, mechanical and thermal properties. Ind. Crop. Prod..

[30] Hablot E., Zheng D., Bouquey M., Avérous L. (2008). Polyurethanes Based on Castor Oil: Kinetics, Chemical, Mechanical and Thermal Properties. Macromol. Mater. Eng..

[31] Desroches M., Escouvois M., Auvergne R., Caillol S., Boutevin B. (2012). From Vegetable Oils to Polyurethanes: Synthetic Routes to Polyols and Main Industrial Products. Polym. Rev..

[32] Ogunniyi D.S. (2006). Castor oil: A vital industrial raw material. Bioresour. Technol..

[33] Mutlu H., Meier M.A.R. (2010). Castor oil as a renewable resource for the chemical industry. Eur. J. Lipid Sci. Technol..

[34] Gebremariam S., Marchetti J. (2018). Economics of biodiesel production: Review. Energy Convers. Manag..

[35] Harmer M.A., Confer D.C., Hoffman C.K., Jackson S.C., Liauw A.Y., Minter A.R., Murphy E.R., Spence R.E., Sunkara H.B. (2010). Renewably sourced polytrimethylene ether glycol by superacid catalyzed condensation of 1,3-propanediol. Green Chem..

[36] Sonnenschein M.F. (2014). Polyurethane Building Blocks. Polyurethanes.

[37] Thomas J., Singh V., Jain R. (2020). Synthesis and characterization of solvent free acrylic copolymer for polyurethane coatings. Prog. Org. Coat..

[38] Kasprzyk P., Datta J. (2018). Effect of molar ratio [NCO]/[OH] groups during prepolymer chains extending step on the morphology and selected mechanical properties of final bio-based thermoplastic poly(ether-urethane) materials. Polym. Eng. Sci..

[39] Kasprzyk P., Sadowska E., Datta J. (2019). Investigation of Thermoplastic Polyurethanes Synthesized via Two Different Prepolymers. J. Polym. Environ..

[40] More A.S., Lebarbé T., Maisonneuve L., Gadenne B., Alfos C., Cramail H. (2013). Novel fatty acid based di-isocyanates towards the synthesis of thermoplastic polyurethanes. Eur. Polym. J..

[41] Tawade B., Shingte R., Kuhire S., Sadavarte N., Garg K., Maher D., Ichake A., More A., Wadgaonkar P. (2017). Bio-based di/ polyisocyanates for polyurethanes: An overview. Polyurethanes Today.

[42] Kamal M.R., Kuder R.C. (1972). Diisocyanates. U.S. Patent.

[43] Chiacchiarelli L.M., Fortunati E., Jain S., Zhang X. (2019). 8-Sustainable, nanostructured, and bio-based polyurethanes for energy-efficient sandwich structures applied to the construction industry. Biomass, Biopolymer-Based Materials, and Bioenergy, Verma, D..

[44] Bishop T.E., Coady C.J., Zimmerman J.M. (1986). Ultraviolet Curable Buffer Coatings for Optical Glass Fiber Based on Long Chain Oxyalkylene Diamines. U.S. Patent.

[45] Coady C.J., Krajewski J.J., Bishop T.E. (1986). Polyacrylated Oligomers in Ultraviolet Curable Optical Fiber Coatings. U.S. Patent.

[46] Charlon M., Heinrich B., Matter Y., Couzigné E., Donnio B., Avérous L. (2014). Synthesis, structure and properties of fully biobased thermoplastic polyurethanes, obtained from a diisocyanate based on modified dimer fatty acids, and different renewable diols. Eur. Polym. J..

[47] Das S., Pandey P., Mohanty S., Nayak S.K. (2015). Influence of NCO/OH and transesterified castor oil on the structure and properties of polyurethane: Synthesis and characterization. Mater. Express.

[48] Sahoo S., Kalita H., Mohanty S., Nayak S. (2017). Synthesis and Characterization of Vegetable oil based Polyurethane derived from Biobased isocyanate. J. Polym. Mater..

[49] Wang C., Cao X., Zhang Y. (2017). A novel bioactive osteogenesis scaffold delivers ascorbic acid, β-glycerophosphate, and dexamethasone in vivo to promote bone regeneration. Oncotarget.

[50] Hao H., Shao J., Deng Y., He S., Luo F., Wu Y., Li J., Tan H., Li J., Fu Q. (2016). Synthesis and characterization of biodegradable lysine-based waterborne polyurethane for soft tissue engineering applications. Biomater. Sci..

[51] Guelcher S.A., Srinivasan A., Dumas J.E., Didier J.E., McBride S., Hollinger J.O. (2008). Synthesis, mechanical properties, biocompatibility, and biodegradation of polyurethane networks from lysine polyisocyanates. Biomaterials.

[52] Storey R.F., Wiggins J.S., Puckett A.D. (1994). Hydrolyzable poly(ester-urethane) networks from L-lysine diisocyanate and D,L-lactide/ε-caprolactone homo- and copolyester triols. J. Polym. Sci. Part. A Polym. Chem..

[53] Lee S.-H., Wang S. (2006). Biodegradable polymers/bamboo fiber biocomposite with bio-based coupling agent. Compos. Part. A Appl. Sci. Manuf..

[54] Liu H., Chen N., Shan P., Song P., Liu X., Chen J. (2019). Toward Fully Bio-based and Supertough PLA Blends via in Situ Formation of Cross-Linked Biopolyamide Continuity Network. Macromolecules.

[55] Acik G., Karabulut H.R.F., Altinkok C., Karatavuk A.O. (2019). Synthesis and characterization of biodegradable polyurethanes made from cholic acid and l-lysine diisocyanate ethyl ester. Polym. Degrad. Stab..

[56] Acik G., Kamaci M., Altinkok C., Karabulut H.F., Tasdelen M.A. (2018). Synthesis and properties of soybean oil-based biodegradable polyurethane films. Prog. Org. Coat..

[57] Cifarelli A., Boggioni L., Vignali A., Tritto I., Bertini F., Losio S. (2021). Flexible Polyurethane Foams from Epoxidized Vegetable Oils and a Bio-Based Diisocyanate. Polymers.

[58] Sahoo S., Kalita H., Mohanty S., Nayak S.K. (2017). Meticulous study on curing kinetics of green polyurethane-clay nanocomposite adhesive derived from plant oil: Evaluation of decomposition activation energy using TGA analysis. J. Macromol. Sci. Part. A.

[59] Gurunathan T., Chung J.S. (2016). Physicochemical Properties of Amino–Silane-Terminated Vegetable Oil-Based Waterborne Polyurethane Nanocomposites. ACS Sustain. Chem. Eng..

[60] Hu J., Chen Z., He Y., Huang H., Zhang X. (2017). Synthesis and structure investigation of hexamethylene diisocyanate (HDI)-based polyisocyanates. Res. Chem. Intermed..

[61] Widemann M., Driest P.J., Orecchia P., Naline F., Golling F.E., Hecking A., Eggert C., Pires R., Danielmeier K., Richter F.U. (2018). Structure–Property Relations in Oligomers of Linear Aliphatic Diisocyanates. ACS Sustain. Chem. Eng..

[62] Kurańska M., Cabulis U., Auguścik M., Prociak A., Ryszkowska J., Kirpluks M. (2016). Bio-based polyurethane-polyisocyanurate composites with an intumescent flame retardant. Polym. Degrad. Stab..

[63] Sasaki N., Yokoyama T., Tanaka T. (1973). Properties of isocyanurate-type crosslinked polyurethanes. J. Polym. Sci. Polym. Chem. Ed..

[64] Levita G., De Petris S., Marchetti A., Lazzeri A. (1991). Crosslink density and fracture toughness of epoxy resins. J. Mater. Sci..

[65] Schroeder J.A., Madsen P.A., Foister R.T. (1987). Structure/property relationships for a series of crosslinked aromatic/aliphatic epoxy mixtures. Polymers.

[66] Lapprand A., Boisson F., Delolme F., Méchin F., Pascault J.-P. (2005). Reactivity of isocyanates with urethanes: Conditions for allophanate formation. Polym. Degrad. Stab..

[67] Olejnik A., Gosz K., Piszczyk L. (2020). Kinetics of cross-linking processes of fast-curing polyurethane system. Thermochim. Acta.

[68] Vyazovkin S., Burnham A.K., Favergeon L., Koga N., Moukhina E., Pérez-Maqueda L.A., Sbirrazzuoli N. (2020). ICTAC Kinetics Committee recommendations for analysis of multi-step kinetics. Thermochim. Acta.

[69] Rochery M., Lam T.M. (2000). Chemorheology of polyurethane. I. Vitrification and gelation studies. J. Polym. Sci. Part. B Polym. Phys..

[70] Kasprzyk P., Benes H., Donato R.K., Datta J. (2020). The role of hydrogen bonding on tuning hard-soft segments in bio-based thermoplastic poly(ether-urethane)s. J. Clean. Prod..

[71] Hassan M.K., Mauritz K.A., Storey R.F., Wiggins J.S. (2006). Biodegradable aliphatic thermoplastic polyurethane based on poly(ε-caprolactone) and L-lysine diisocyanate. J. Polym. Sci. A Polym. Chem..

