# Supplementary material for: Fully Bio-Based Thermosetting Polyurethanes from Bio-Based Polyols and Isocyanates
Source: Polymers (Basel). 2021 Apr 13;13(8):1255. doi: 10.3390/polym13081255 (PMC8069015; doi:10.3390/polym13081255)
Supplement: Supplementary file 1 [file polymers-13-01255-s001.pdf]

Fully bio-based thermosetting polyurethanes from bio-based polyols and isocyanates

Roberto MORALES-CERRADA[‡1], Romain TAVERNIER[‡1], Sylvain CAILLOL[1*]

[1] ICGM, Univ Montpellier, CNRS, ENSCM, Montpellier, France

*Corresponding author: sylvain.caillol@enscm.fr

[‡]These authors have equally contributed.

Supplementary material

1. Experimental procedures

Table S1. Experimental polyisocyanate and castor oil amounts of entries L1, T1 and D1.

Entry	Polyisocyanate	Polyisocyanate amount (g)	Castor oil amount (g)
L1	LDI	2.57	7.43
T1	Tolonate™ X FLO 100	5.04	4.96
D1	Desmoudur® eco N 7300	3.42	6.58

Table S2. Experimental polyisocyanate, Velvetol® H500 and chain extender amounts of entries L2, T2 and D2.

Entry	Polyisocyanate	Polyisocyanate amount (g)	Velvetol® H500 amount (g)	Calculated prepolymer IEW (g·eq^{-1})	Chain extender	Chain extender amount (g)
L2	LDI	5.30	4.70	415	Glycerol	0.75
T2	Tolonate™ X FLO 100	7.68	2.32	820	Glycerol	0.38
D2	Desmoudur® eco N 7300	7.18	2.82	348	1,3-Propanediol	1.12

Table S3. Experimental polyisocyanate, Velvetol® H500 and glycerol amounts of entries L3, T3 and D3.

Entry	Polyisocyanate	Polyisocyanate amount (g)	Velvetol® H500 amount (g)	Glycerol amount (g)
L3	LDI	3.20	6.46	0.34
T3	Tolonate™ X FLO 100	5.81	3.98	0.21
D3	Desmoudur® eco N 7300	4.05	5.95	0

2. ESI-MS

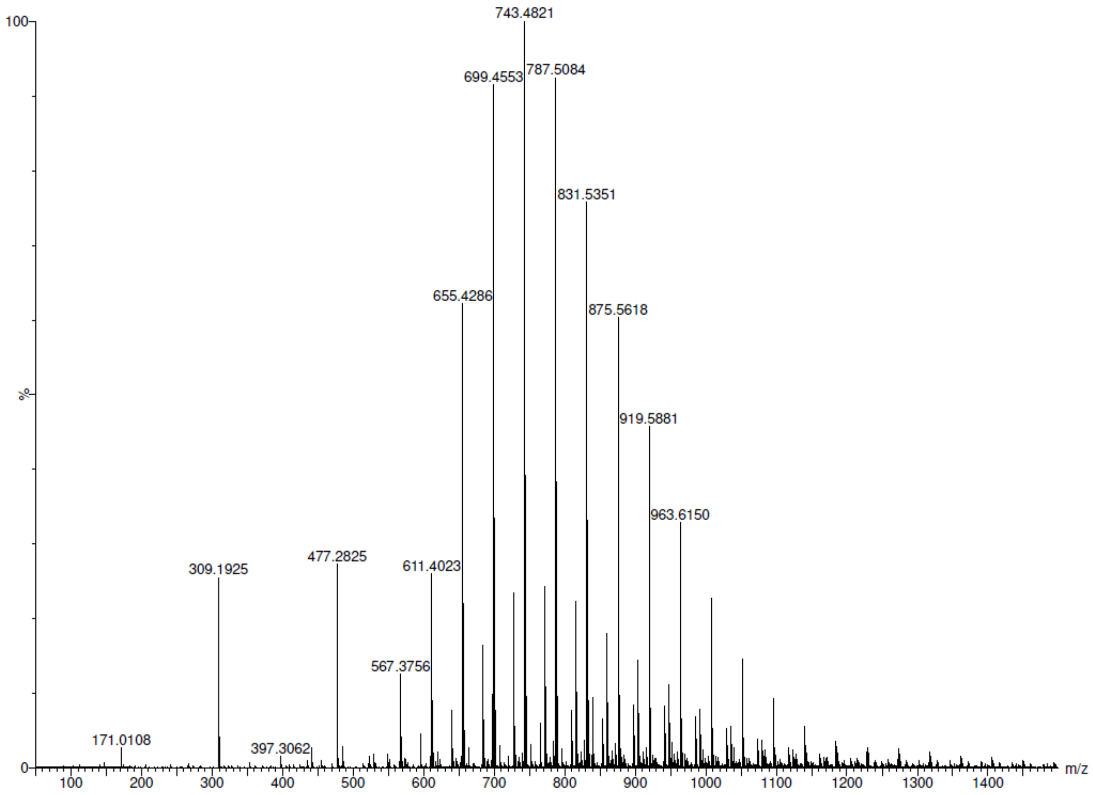

Figure S1. ESI-MS spectra (negative) of Tolonate X FLO 100 in methanol.

3. ^{1}H NMR spectroscopy

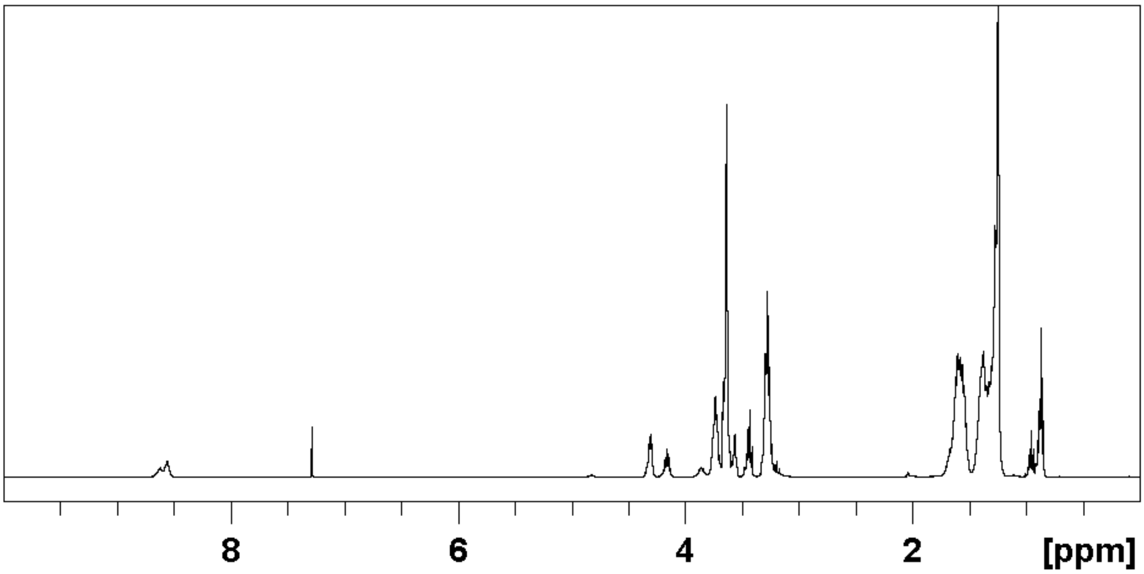

Figure S2. ^{1}H NMR spectrum (400 MHz, $CDCl_3$) of Tolonate™ X FLO 100.

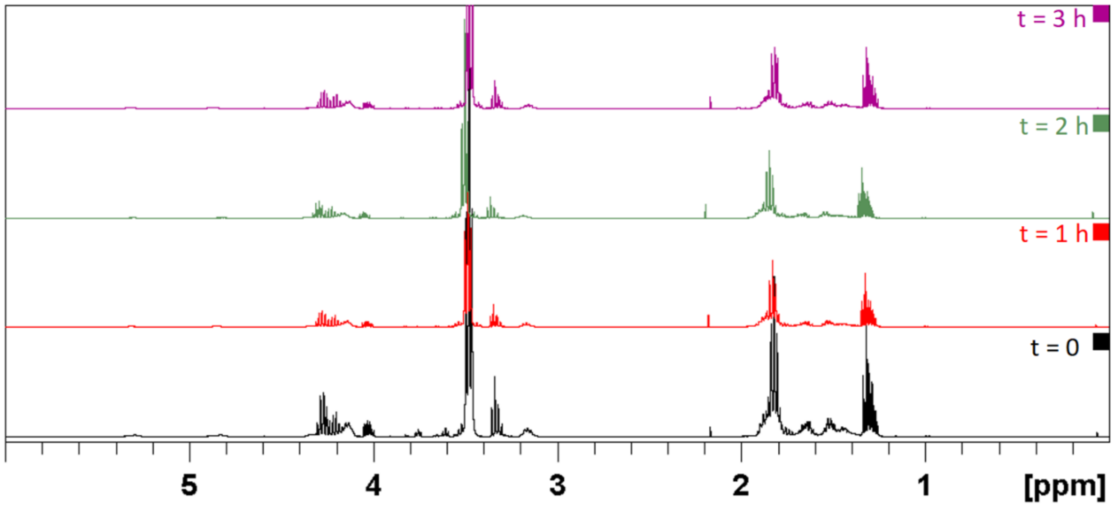

Figure S3. ^{1}H NMR spectra (400 MHz, $CDCl_3$) of LDI and Velvetol® H500 reaction at 0, 1, 2 and 3 h after the complete addition of Velvetol® H500.

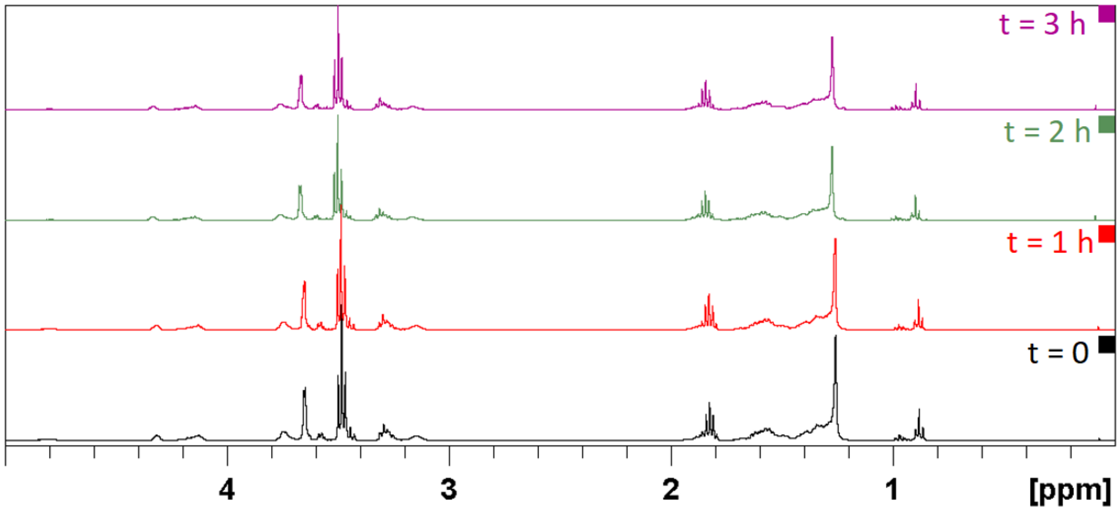

Figure S4. ^{1}H NMR spectra (400 MHz, $CDCl_3$) of Tolonate™ X FLO 100 and Velvetol® H500 reaction at 0, 1, 2 and 3 h after the complete addition of Velvetol® H500.

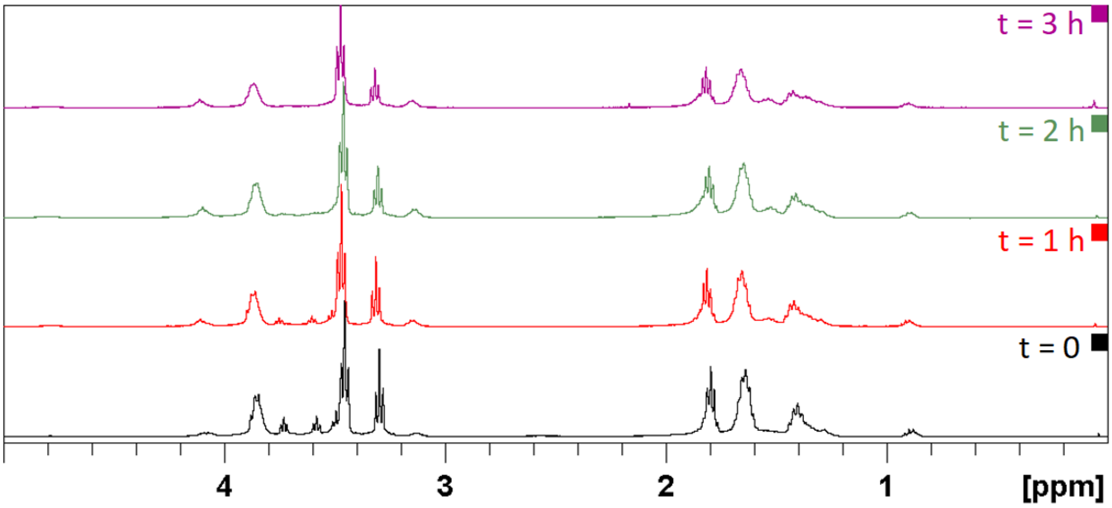

Figure S5. ^{1}H NMR spectra (400 MHz, $CDCl_3$) of Desmodur® eco N 7300 and Velvetol® H500 reaction at 0, 1, 2 and 3 h after the complete addition of Velvetol® H500.

4. Differential Scanning Calorimetry

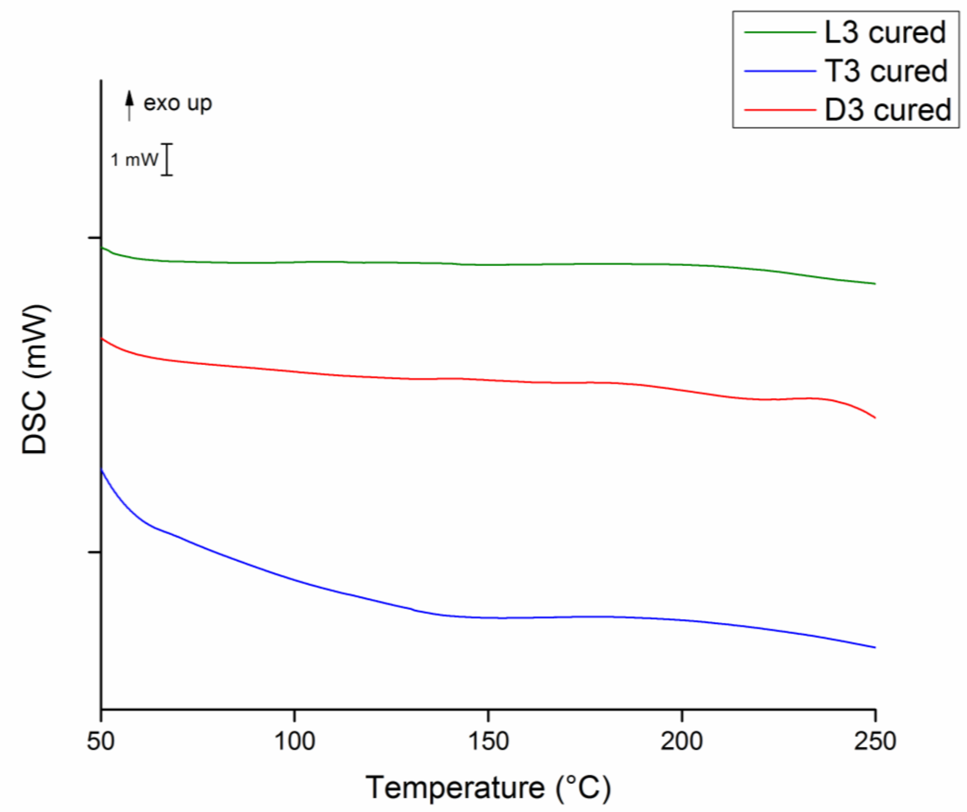

Figure S6. DSC of one-pot formulations L3, T3 and D3 with Velvetol H500 at 40 °C·min^{-1} after curing at 80 °C for 24h.

5. Rheology

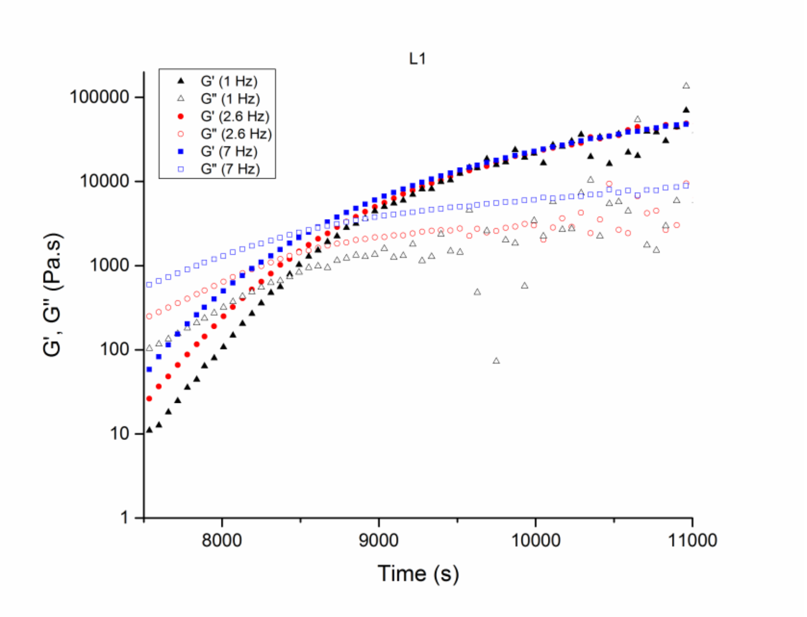

Figure S7. Gelation time determination for L1 at 80 °C.

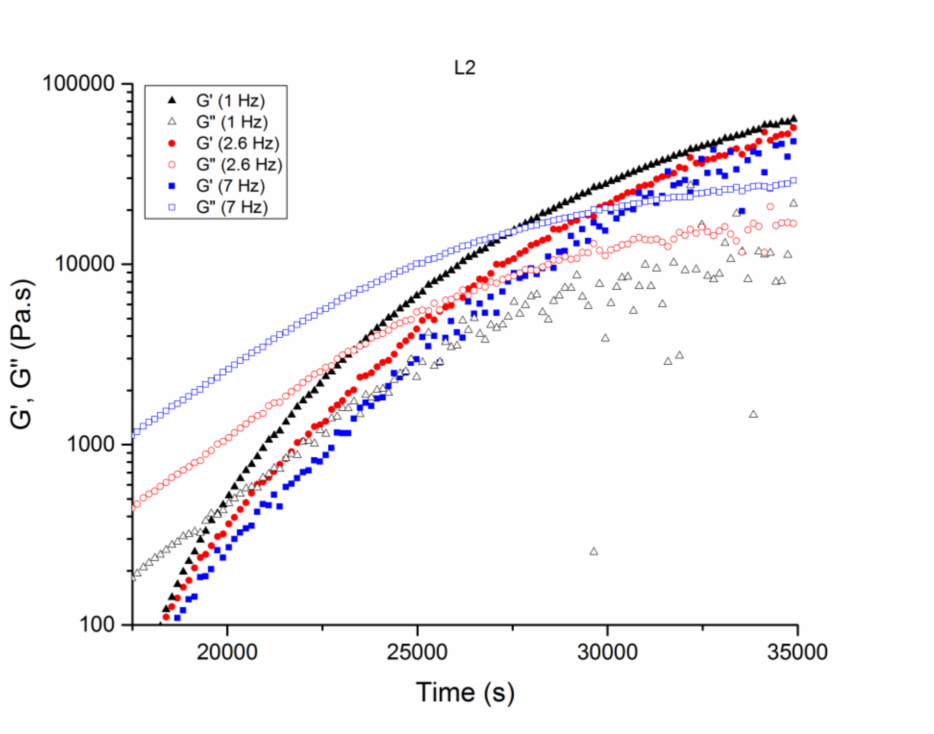

Figure S8. Gelation time determination for L2 at 80 °C.

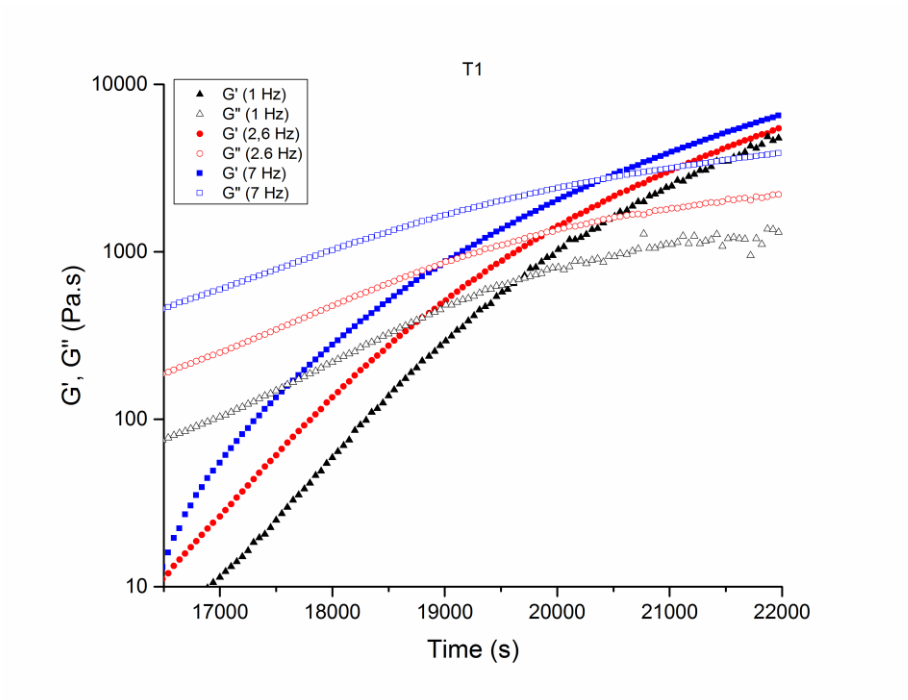

Figure S9. Gelation time determination for T1 at 80 °C.

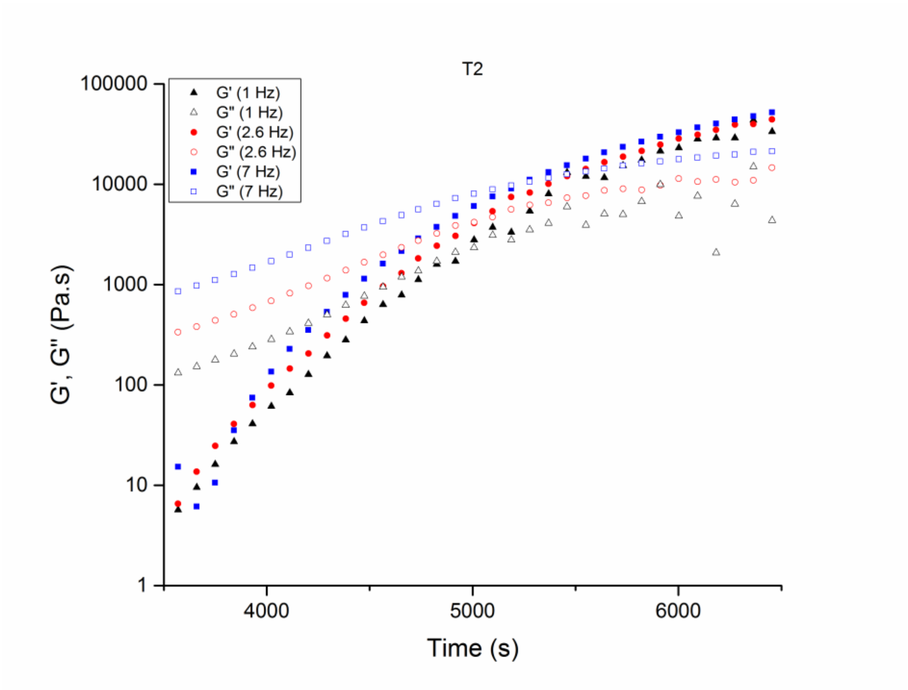

Figure S10. Gelation time determination for T2 at 80 °C.

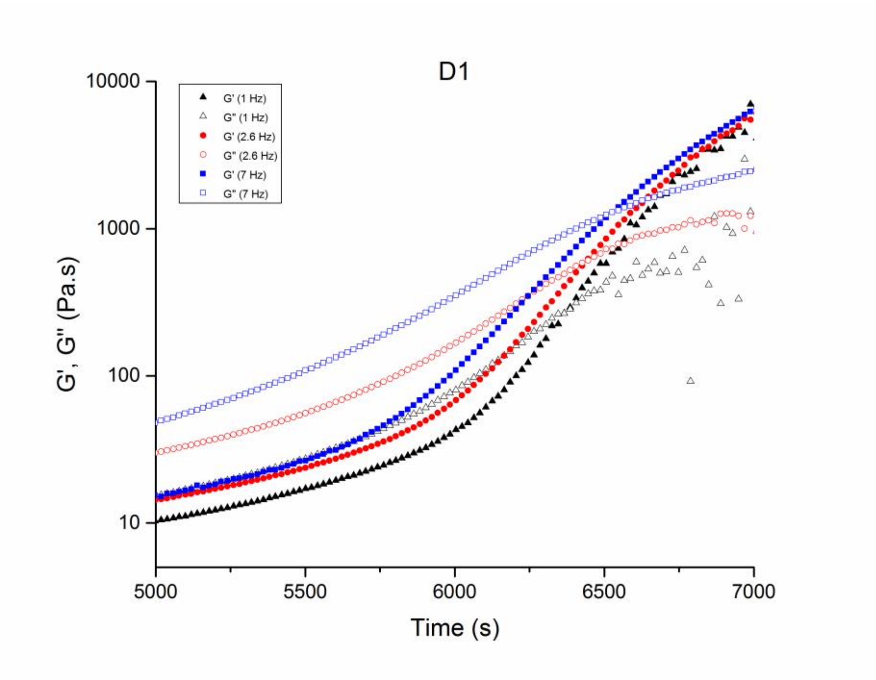

Figure S11. Gelation time determination for D1 at 80 °C.

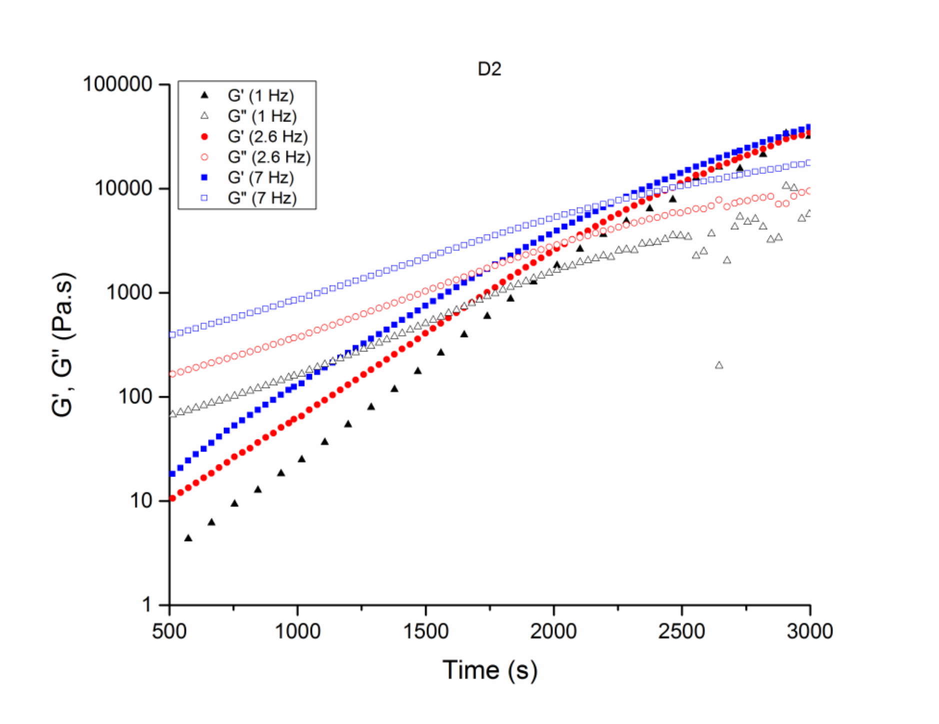

Figure S12. Gelation time determination for D2 at 80 °C.

6. FTIR spectroscopy

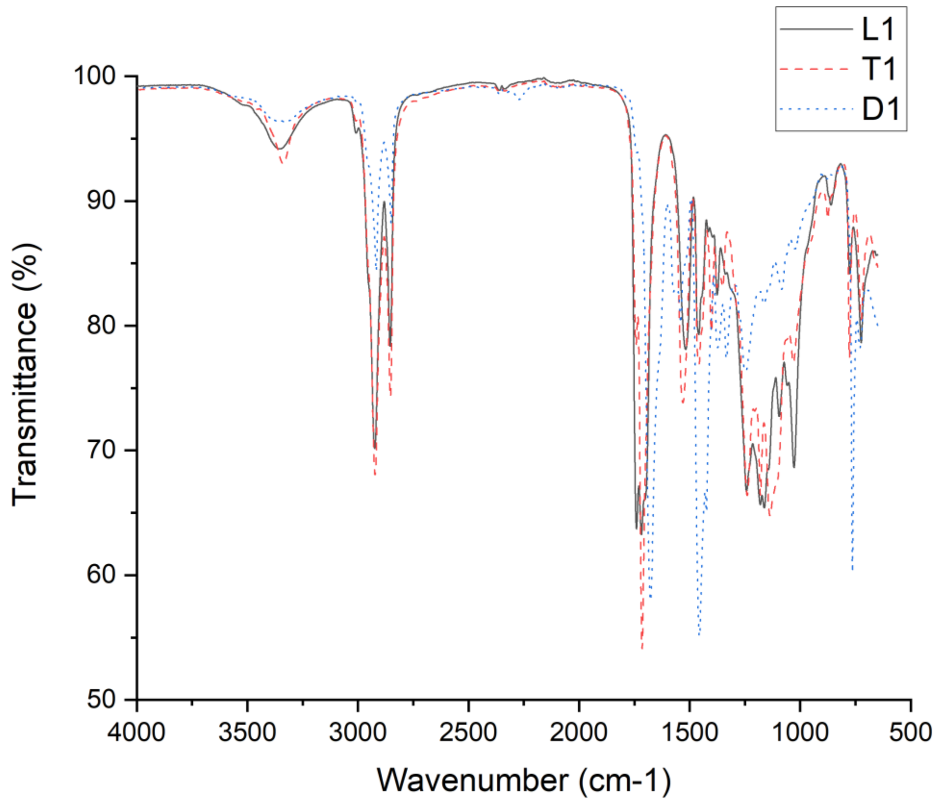

Figure S13. FTIR spectra (ATR) of cured materials of entries L1, T1 and D1.

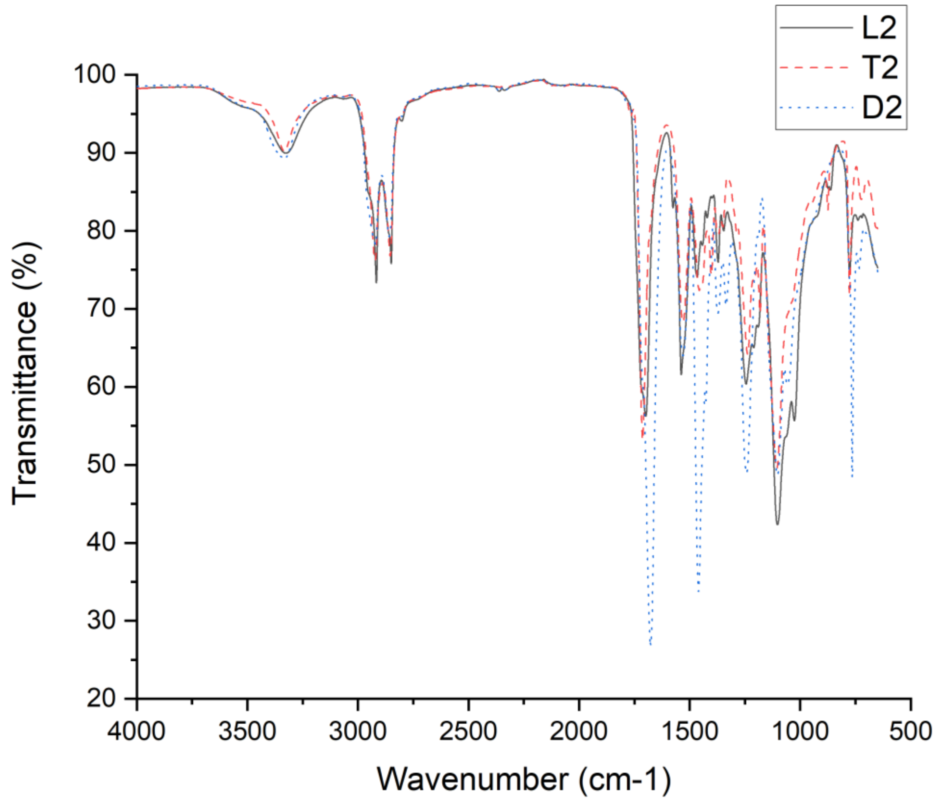

Figure S14. FTIR spectra (ATR) of cured materials of entries L2, T2 and D2.

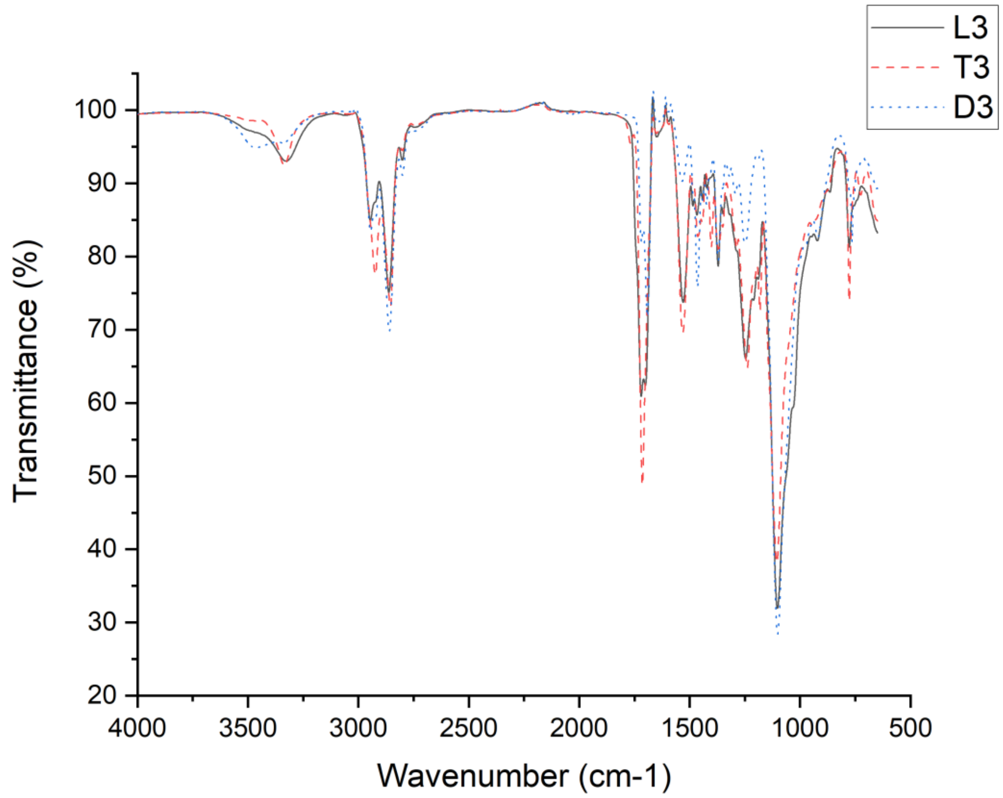

Figure S15. FTIR spectra (ATR) of cured materials of entries L3, T3 and D3.

7. TGA

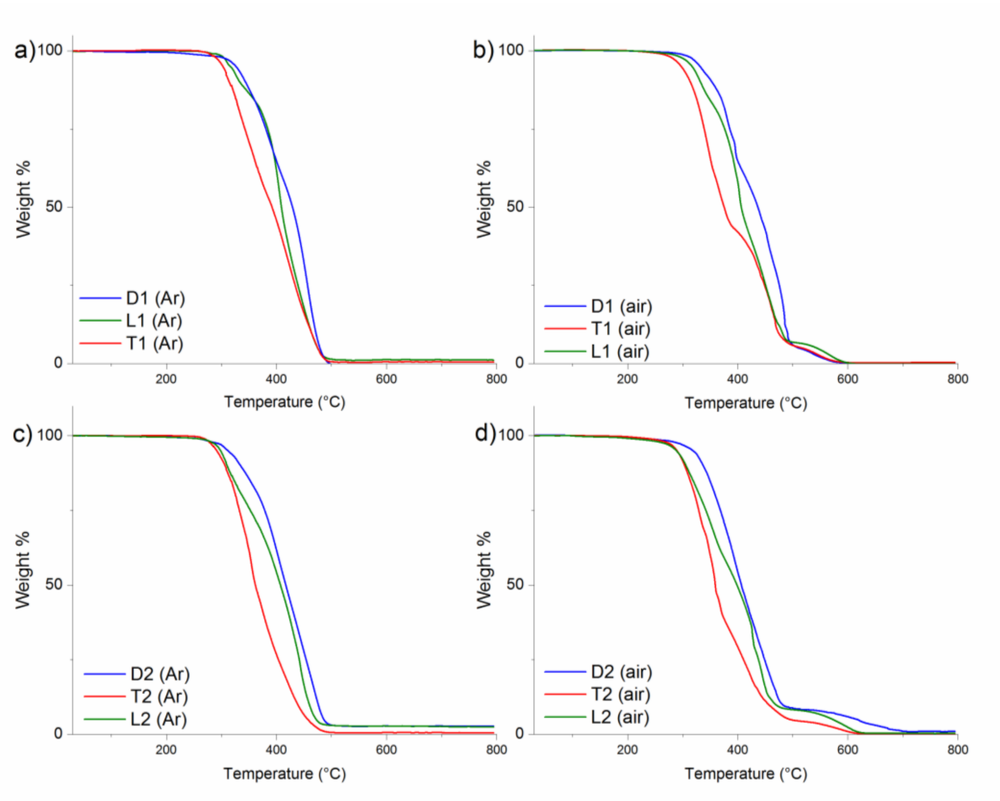

Figure S16 TGA (20 °C·min-1) of cross-linked polyurethane formulations - a) one-step formulations, under Argon - b) one-step formulations, under air - c) two-steps formulations under Argon and d) two-steps formulations under air.
